# Early mucosal IFN-α, IP-10, and IL-1RA and synchronized mucosal and systemic immune responses mediate COVID-19 disease progression

**DOI:** 10.1128/mbio.01491-25

**Published:** 2025-11-28

**Authors:** Mona Agrawal, Armando S. Flores-Torres, John S. Franks, Sarah Y. Lang, Thomas P. Fabrizio, Kristin E. McNair, Laura V. Boywid, Ashley J. Blair, Chloe N. Hundman, Nicholas D. Hysmith, Michael A. Whitt, Rachael Keating, Paul G. Thomas, Richard J. Webby, Amanda M. Green, Heather S. Smallwood

**Affiliations:** 1 Department of Pediatrics, The University of Tennessee Health Science Center 12326, Memphis, Tennessee, USA; 2 Department of Infectious Disease, St Jude Children's Research Hospital 5417, Memphis, Tennessee, USA; 3 Department of Microbiology, Immunology & Biochemistry, The University of Tennessee Health Science Center 12326, Memphis, Tennessee, USA; 4 Department of Pediatrics, Le Bonheur Children’s Hospital, Memphis, Tennessee, USA; 5 Children’s Foundation Research Institute, Memphis, Tennessee, USA; 6 Department of Anatomy and Developmental Biology, Monash University 195115, Clayton, Victoria, Australia; 7 Department of Host Microbe Interactions, St Jude Children’s Research Hospital, Memphis, Tennessee, USA; Monash University, Clayton, Victoria, Australia

**Keywords:** immune mechanisms, immune response, mucosal immunity, viral immunity, viral clearance, viral pathogenesis, infectious disease, pediatric infectious disease, humoral immunity, COVID-19, medical outcomes, cytokines

## Abstract

Mucosal immunity plays a crucial role in protection against respiratory viruses. However, the mechanisms underlying mucosal responses and their impact on COVID-19 outcomes are not well understood, as mucosal immunity is compartmentalized and not always reflected in the bloodstream. This study examined primary immune responses in 584 mucosal and blood specimens collected over a month from previously naïve adults and children with COVID-19. Various laboratory techniques were utilized to quantify and characterize viral RNA, antigens, antibodies, and cytokines in the samples, including PCR, sequencing, ELISA, and Luminex. Comprehensive system analysis uncovered distinctive characteristics associated with mild COVID-19 disease progression, including markedly early and elevated induction of mucosal IFN-α and IP-10, followed by increased levels of IL-1RA and IgG. Individuals experiencing mild COVID-19 demonstrated synchronized mucosal and systemic immune responses, with a gradual increase in antibody production that resulted in enhanced neutralization potency, potentially conferring greater protection against future infection. In contrast, individuals with moderate and severe COVID-19 exhibited diminished IFN-α and IP-10 responses and dysregulated mucosal and systemic immune responses marked by rapid and robust yet less effective humoral immunity, potentially driven by high antigen and cytokine levels in both compartments. Collectively, these findings underscore that early mucosal immune responses may play a pivotal role in attenuating COVID-19 disease severity. Additionally, they suggest that primary mucosal immune responses to novel viruses influence clinical outcomes, providing critical insights necessary for developing prognostic indicators, treatments, and mucosal vaccines that confer protection against SARS-CoV-2 and emerging respiratory pathogens.

**IMPORTANCE:**

This research is crucial for understanding the intricate interplay between mucosal immunity and SARS-CoV-2 infection. By examining the distinct systemic and mucosal immune responses during COVID-19, this study addresses the critical gap in our knowledge of how the body defends itself at the primary site of infection: the respiratory mucosa. The findings shed light on the specific characteristics of the mucosal immune response, including the roles of different antibody isotypes, immune cells, and local factors in controlling viral entry and replication. Furthermore, because this study focuses on *de novo* immune responses, the results may have broad implications for understanding immune responses to future novel pathogens. Ultimately, this research will contribute to the development of more effective diagnostic tools, therapeutic strategies, and mucosal vaccines to prevent and control COVID-19. By focusing on the often-overlooked mucosal compartment, this work offers a new perspective on comprehending SARS-CoV-2 infection, its implications for public health, and preparedness for future pandemic threats.

## INTRODUCTION

COVID-19 remains a public health threat, and the prospect of another novel respiratory pathogen emerging looms. Immunity from infection and vaccination has reduced disease severity, yet the wide clinical spectrum of COVID-19 remains an enigma. For reasons that remain unclear, African Americans (AA) and children were observed to be at higher and lower risk for poor outcomes, respectively (1–3). Viral load does not account for these disparate outcomes. Viral load was lower in severity-matched AA compared to Americans of European descent (EA), and it was similar among pediatric and adult patients (4, 5). SARS-CoV-2 nucleocapsid (N) and spike (S) proteins are highly antigenic and present in the mucosa (6, 7), yet the bulk of our knowledge is based on systemic S-specific antibody responses, which were elevated in severe COVID-19 (8–10). Overexuberant systemic cytokine responses were also associated with severe COVID-19 (11, 12), with the magnitude of systemic cytokines contributing to the wide range of COVID-19 outcomes (13). However, in 2020, computational modeling showed that adaptive responses preceded the peak of local infection in severe COVID-19 cases (14), suggesting that mucosal responses may play a role in systemic immune activation and disease progression.

Mucosal responses control viral replication, lung invasion, and induction of immune activation (15, 16). Mucosal interferon (IFN) levels inversely correlated with viral load and positively correlated with peripheral antibodies (17, 18). More recent clinical studies found that viral load had no impact on the speed of recovery from COVID-19, whereas increased early mucosal antibodies accelerated it (19, 20), suggesting that they were critical for timely clearance. Higher levels of mucosal antibodies were also associated with lower risk of future SARS-CoV-2 infections (21). Single-cell profiling of nasal swabs from naïve individuals challenged with SARS-CoV-2 showed that asymptomatic, transient infection (*n* = 3) was associated with early immune cell infiltration, whereas symptomatic individuals (*n* = 6) had delayed responses with sustained viral replication (22). Despite small sample size, these data suggest that early mucosal responses may be crucial for fighting novel viruses including SARS-CoV-2. Nonetheless, the majority of clinical studies lack cohort diversity, asymptomatic and mild illness, and nasopharyngeal sampling, thereby limiting our understanding of mucosal immune responses to SARS-CoV-2 (23, 24).

Reducing the health burden of COVID-19 and future emerging respiratory viruses requires improving our understanding of mucosal immunity and its association with peripheral responses and disease progression in the general population. Here, we investigated the levels of SARS-CoV-2 RNA, S and N proteins, antigen-specific antibodies, and cytokines in swabs, nasopharyngeal rinses, and blood over one month from a diverse cohort of previously naive participants and assessed how these factors relate to protection against a novel viral infection and contribute to clinical outcomes.

## RESULTS

### Population and specimens

**Fig 1:**
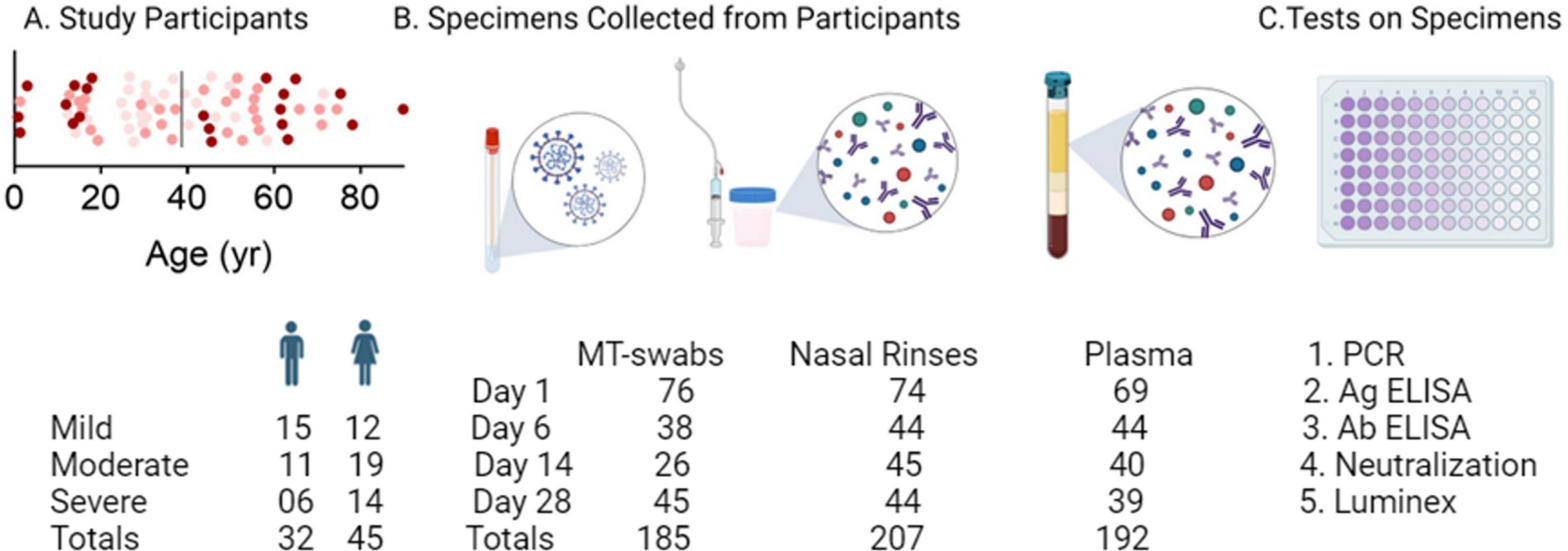
Characteristics of study participants, specimen, and tests to determine mucosal and systemic viral and immune dynamics. (**A**) Age and sex distribution of subjects by COVID-19 severity; mild (pink), moderate (salmon), or severe (maroon). (**B**) Schematic representation of mid turbinate swabs, nasal rinses, and plasma collected over one month, with the number of participants from whom the samples were collected. (**C**) The experimental procedures performed on specimens included PCR on swabs, neutralization on plasma, antigen and antibody by ELISA, and cytokines by Luminex on both nasal fluids and plasma.

To assess primary immune responses to a novel respiratory viral infection, 77 participants, enrolled between July 15, 2020, to March 9, 2021, were classified as having mild (*n* = 27), moderate (*n* = 30), or severe (*n* = 20) COVID-19 (Table 1). Demographics and clinical data are summarized in Table 2. Subjects were equally distributed among severity groups. We used 584 longitudinally collected mucosal and blood samples in our analysis (Fig. 1).

**TABLE 1:** Severity scoring criteria^*a*^

Criteria groups	Point(s)
**Mild symptoms:** cough, fever, diarrhea, vomiting, headache, loss of taste or smell, sore throat, myalgias, fatigue, lymphadenopathy, and malaise	1
**Moderate symptoms:** shortness of breath (dyspnea), wheezing, SpO_2_ <92% on room air, respiratory rate (RR) >30, and new non-invasive oxygen requirement	1
**Severe symptoms:** invasive or positive-pressure oxygen requirement, acute kidney injury (C*r >*1.5× upper limit normal for age or estimated glomerular filtration rate [eGFR] <60), elevated aspartate/alanine transaminase (AST/ALT) ratio >2× normal, new elevation international normalized ratio (INR) >1.3, and altered mental status	2
**Critical symptoms:** acute respiratory distress syndrome (ARDS), shock requiring pressors, renal failure with dialysis, extracorporeal membrane oxygenation (ECMO) requirement, requirement of organ transplant, pulmonary embolism, deep venous thrombosis, and stroke	3
**Hospital admission:** within 60 days of enrollment	2
**ICU admission:** within 60 days of enrollment	3

^*a*^ Participant severity outcomes were classified as mild, moderate, or severe COVID-19 based on cumulative point scores of ≤1, 2–4, or ≥5, respectively.

**TABLE 2:** Demographics, symptoms, and clinical characteristics of the cohort^*a*^

Characteristics	Mild *N* (%)	Moderate *N* (%)	Severe *N* (%)	Total *N* (%)	*P*
Total subjects					
COVID-19 positive	27 (35)	30 (39)	20 (26)	77 (100)	0.22
Age groups					
Child (≤19 yrs)	3 (3.9)	8 (10.4)	8 (10.4)	19 (24.7)	0.15
Adult (20 to 65 yrs)	23 (29.9)	18 (23.4)	8 (10.4)	49 (63.6)	**<0.01**
Senior (>65 yrs)	1 (1.3)	4 (5.2)	4 (5.2)	9 (11.7)	0.22
Sex groups					
Male	15 (19.5)	11 (14.3)	6 (7.8)	32 (41.6)	0.06
Female	12 (15.6)	19 (24.7)	14 (18.2)	45 (58.4)	0.27
Racial groups					
AA	1 (1.3)	12 (15.6)	14 (18.2)	27 (35)	**<0.01**
EA	10 (12.9)	12 (15.6)	6 (7.8)	28 (36.4)	0.22
Unknown	16 (20.8)	6 (7.8)	0 (0)	22 (28.6)	**<0.01**
Age (median [IQR])					
Total	30.4 (43.7)	46.4 (55.4)	45.3 (62.5)	37.2 (55.3)	0.43
Child	14.2 (15.4)	16 (17.6)	15.4 (11.6)	14.8 (16.7)	0.57
Adult	32 (43.8)	49.1 (54.7)	59.9 (62)	43.8 (51.5)	**<0.01**
Senior	71.3 (71.3)	70.9 (72)	76.8 (81.2)	71.4 (75.4)	0.49
Symptoms					
Upper respiratory	15 (55.6)	16 (53.3)	5 (0.25)	36 (46.8)	0.08
Lower respiratory	12 (44.4)	23 (76.7)	16 (80.0)	51 (66.2)	**0.01**
Anosmia	15 (55.6)	13 (43.3)	1 (5.0)	29 (37.7)	**<0.01**
Chest pain	0 (0)	7 (23.3)	8 (40.0)	15 (19.5)	**<0.01**
Gastrointestinal	4 (14.8)	15 (50.0)	9 (45.0)	28 (36.4)	**0.01**
Systemic	12 (44.4)	20 (66.7)	16 (80.0)	48 (62.3)	**0.04**
Comorbidities					
Respiratory disease	1 (3.7)	7 (23.3)	9 (45.0)	17 (22.1)	**<0.01**
Heart disease	2 (7.4)	8 (26.7)	6 (30.0)	16 (20.8)	0.10
Obesity	3 (11.1)	13 (43.3)	9 (45.0)	25 (32.5)	**0.01**
Hypertension	1 (3.7)	13 (43.3)	10 (50.0)	24 (31.2)	**<0.01**
Diabetes	0 (0)	8 (26.7)	9 (45.0)	17 (22.1)	**<0.01**
Immunosuppressed	0 (0)	3 (10.0)	2 (10.0)	5 (6.5)	0.80
Other conditions	3 (11.1)	13 (43.3)	13 (65.0)	29 (37.7)	**<0.01**
Outcomes					
Hospitalized	0 (0)	16 (20.8)	20 (26)	36 (46.8)	**<0.01**
Survived	27 (100)	30 (100)	18 (90.0)	75 (97.4)	0.10

^*a*^ Coronavirus disease 2019 (COVID-19), African American (AA), European Americans (EA), interquartile range (IQR), intravenous immunoglobulin (IVIG). Race data were self-reported by participants. Continuous variable (age) shown as median and 75th IQR percentiles. One-way ANOVA test done among severity groups to find *P* value. χ test was performed for age, sex, and racial groups (*P* = 0.033, 0.167, and 0.0001, respectively). Outcomes with subgroups was analyzed by Fisher LSD. The relationship between all other categorical variables and severity outcomes were determined with χ test. Values in bold are significant (*P* < 0.05).

### Efficient viral and antigen clearance associated with mild disease progression

All subjects with mild symptoms cleared the virus within a week of diagnosis, whereas 10% and 15% of those who progressed to moderate and severe disease remained PCR positive (Fig. S1A). Individuals with mild or moderate COVID-19 significantly reduced mucosal viral load over time, but this was not the case for those with severe illness (Fig. S1A). Only ancestral B.1 lineages were detected in patients’ swabs, including B.1.223, B.1.369, B.1.2, and related sub-lineages (Fig. S1B). Importantly, no Alpha (B.1.1.7) or other variants of concern were detected during the study period. N and S antigens were detected in the mucosa within one week of diagnosis (acute infection phase); 14% and 48% of subjects were S and N positive, respectively. Systemic S was significantly higher in severe cases (Fig. S1C). N was not detected in plasma. Notably, within a week of diagnosis, class switching from IgM+/IgG- to IgM+/IgG+ was indicative of disease severity. Nearly 50% of participants who developed severe disease, compared to under 10% of those with mild disease, were IgM+/IgG+ (Fig. S1D). By four weeks, approximately 90% of severe and 50% of mild cases were IgM+/IgG+ (Fig. S1D). Severe cases failed to rapidly reduce viral load, exhibited higher antigen levels, and showed faster isotype switching. These data suggest that individuals who rapidly initiate mucosal viral clearance develop mild illness.

### Controlled mucosal and peripheral humoral responses linked to mild disease progression

**Fig 2:**
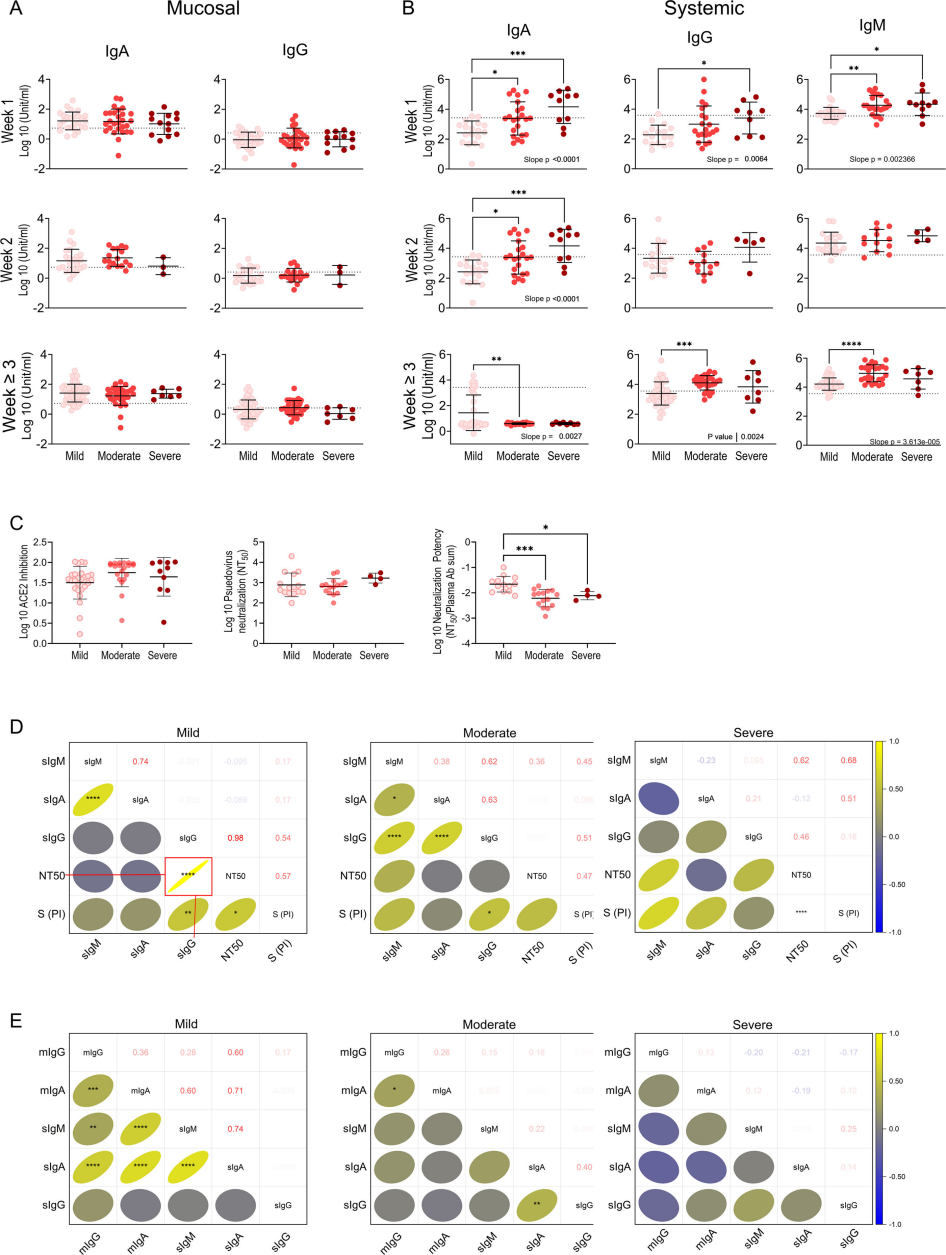
Controlled antibody production and coordination between mucosal and systemic humoral responses are associated with milder disease progression. N- and S-specific antibodies in longitudinally collected NRF and plasma were quantified by ELISA. Each circle represents a patient colored by severity; mild (pink), moderate (salmon), or severe (maroon). The study day was converted to time from positive diagnosis and grouped by week (**A–C**). Mucosal antibodies were quantified in NRF from mild (*n =* 102 samples), moderate (*n =* 77 samples), and severe (*n =* 23 samples) cases (**A**). Systemic antibodies were quantified in plasma from mild (*n =* 86 samples), moderate (*n =* 62 samples), and severe (*n =* 23 samples) cases (**B**). Dotted lines represent the cutoff of positivity. Antibody data were transformed, normal distribution verified, followed by one-way ANOVA with Tukey MCT. Asterisks indicate significant difference in the mean. Post-test for linear trends with increasing severity was performed, *P* values for significant trends inset (**A, B**). Antibody microneutralization was determined in the post-acute phase. Pseudovirus neutralization was quantified in serum from mild (*n =* 15), moderate (*n =* 15), and severe (*n =* 4) cases. Spike neutralization was quantified in plasma samples from mild (*n =* 26), moderate (*n =* 20), and severe (*n =* 10) cases. Antibody neutralization potency was calculated by dividing the NT_50_ by the total antibodies present in samples. Mean difference in severity groups was analyzed by one-way ANOVA with Fisher’s LSD test (**C**). Statistical significance of correlations was determined using Pearson correlation analysis. Asterisks indicate significant correlation, and *r* values are inset in and represented in color bar (**D–E**). The strength of linear correlation between antibody levels, neutralization, and spike protein (S PI) (**D**) and between mucosal and systemic antibodies from 130 paired samples (**E**). Significance levels are indicated by asterisks (*P < 0.05; **P ≤ 0.01; ***P ≤ 0.001; ****P ≤ 0.0001).

To determine the relationship between mucosal and peripheral humoral responses and disease progression, we quantified S- and N-specific antibodies over time. Nasoconversion rates and mucosal IgA levels were high, irrespective of severity (Fig. 2A; Fig. S2A and C). However, individuals with mild to moderate illness significantly increased mucosal IgG production over time (Fig. S2C). Seroconversion rates and systemic IgA, IgG, and IgM levels during week 1 significantly increased as clinical outcomes worsened (Fig. 2B; Fig. S2B). Systemic IgA, IgG, and IgM production steadily increased over time with mild disease (Fig. S2D). Mild cases also displayed a transient increase in IgM that peaked at week 2, reflecting class switching in week 3, yet IgM levels remained elevated in moderate and severe cases, indicating continued antigen recognition and antibody synthesis (Fig. S2D). In recovery, despite similar ACE2 receptor binding inhibition and titers, subjects with mild illness produced antibodies with significantly higher neutralization potency (Fig. 2C). They also exhibited a significant positive correlation between IgG levels and neutralization (Fig. 2D). Only the mild cases had strong and significant positive correlations between local and peripheral humoral responses (Fig. 2E).

### Distinct mucosal and systemic immune response dynamics associated with clinical outcomes

**Fig 3:**
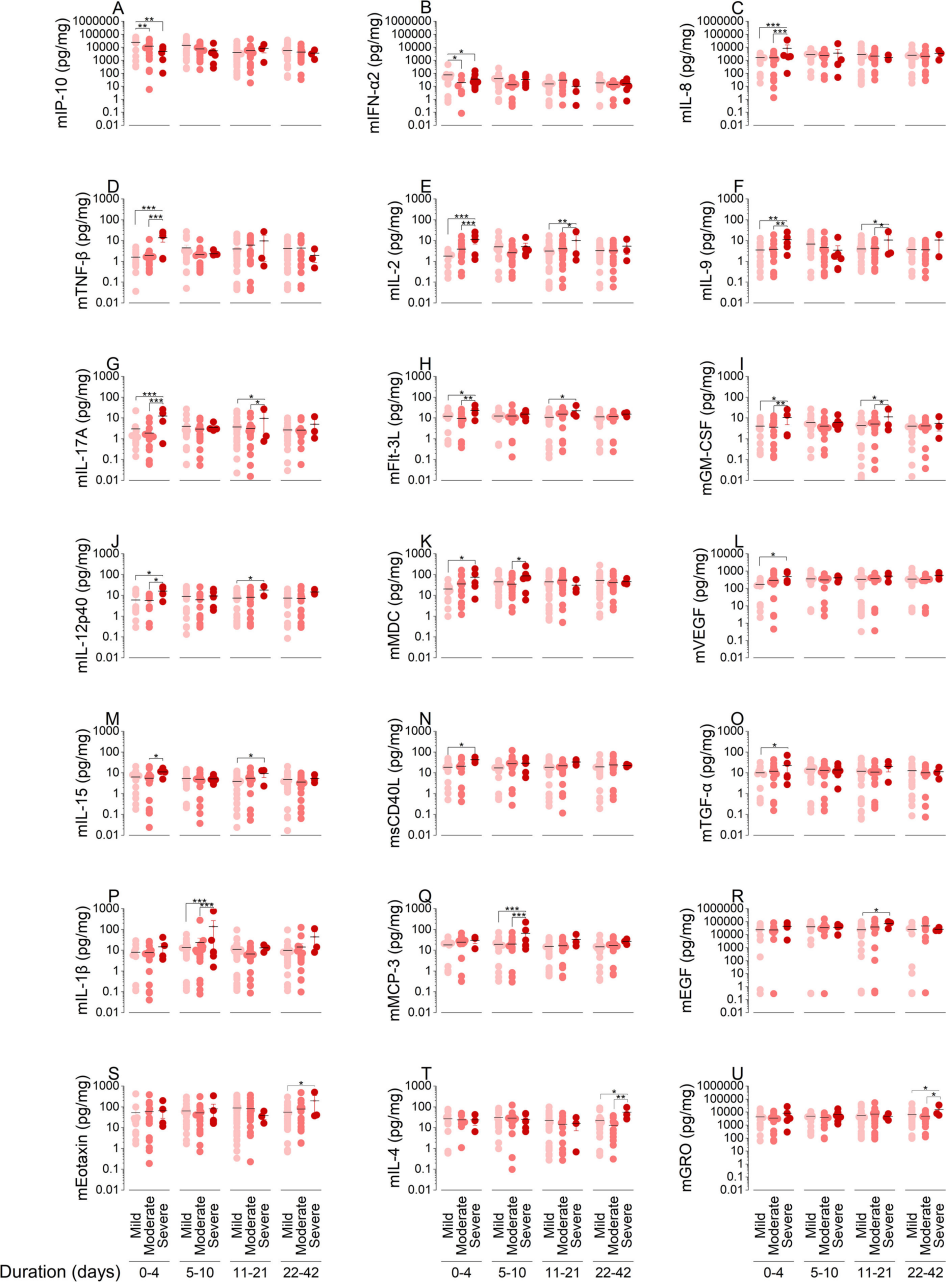
Early mucosal IFN-α and IP-10 are elevated in mild disease progression. Immune factors were quantified on the Luminex xMAP system with a MILLIPLEX MAP human cytokine/chemokine immunoassay (**A–U**). Mucosal cytokine responses were quantified in 66 subjects from mild (*n* = 27), moderate (*n* = 26), and severe (*n* = 13) groups. Each closed circle represents a subject; color indicates mild (pink), moderate (salmon), or severe (maroon) outcome. A total of 190 NRF samples were quantified longitudinally (*n* = 59, 44, 43, and 44 samples collected from study day 1, 6, 14, and 28, respectively). Study day was converted to time from positive diagnosis, and samples were analyzed for early response (0–4 days), adaptive response (5–10 days), resolution (11–21 days), and convalescence (>21 days) phase. Statistical significance of the difference between the means was determined by two-way ANOVA with Tukey’s HSD test (*P < 0.05; **P < 0.01; and ***P < 0.001).

To study how disease progression in previously naïve individuals correlates with mucosal and systemic immunity, we quantified immune factors in NRF and plasma (Fig. S3A and B, respectively). To understand the involvement of immune factors at mucosa and periphery during different stages of viral infection and immune responses, we analyzed immune factors among severity groups during early (0–4 days), adaptive (5–10 days), resolution (11–21 days), and convalescence (>21 days) phases. Individuals who developed mild illness produced significantly more mucosal IFN-α2 and IP-10 during the early response phase (Fig. 3A and B). Patients with moderate to severe COVID-19 significantly increased the production of mucosal pro-inflammatory factors, including IL-8, TNF-β, IL-2, IL-9, IL-17A, Flt-3L, GM-CSF, IL-12p40, MDC, VEGF, IL-15, sCD40L, and TGF-α, during the early response phase (Fig. 3C through O). Severe cases also showed increased levels of mucosal MDC, IL-1β, and MCP-3 during adaptive response phase (Fig. 3K, P and Q), mucosal IL-2, IL-9, IL-17A, Flt-3L, GM-CSF, IL-12p40, IL-15, and EGF during symptoms resolution phase (Fig. 3E through J, M and R) and mucosal eotaxin, IL-4, and GRO during convalescence phase (Fig. 3S through U), compared to mild and moderate cases.

We input these cytokine data into energy flow diagrams that indicate the magnitude of each factor through their convergence on cellular targets (Fig. S4). During the early response phase, those with mild COVID-19 displayed enhanced targeting of T cells, dendritic cells (DC), and natural killer (NK) cells in the upper airways largely via chemokines (Fig. S4A green). The total pg/mg of factors targeting T cells, DCs, and NK cells declined from 28,037 to 15,844 pg/mg in mild versus severe cases, respectively. The most striking evidence of enhanced mucosal antiviral responses was IP-10. Mild cases had twice and almost five times higher levels compared to moderate and severe cases, respectively. Factors targeting endothelial, epithelial, and fibroblast cells totaled 31,117 pg/mg in mild and 56,897 pg/mg in severe cases. This increase suggests airway damage in these patients (Fig. S4A).

**Fig 4:**
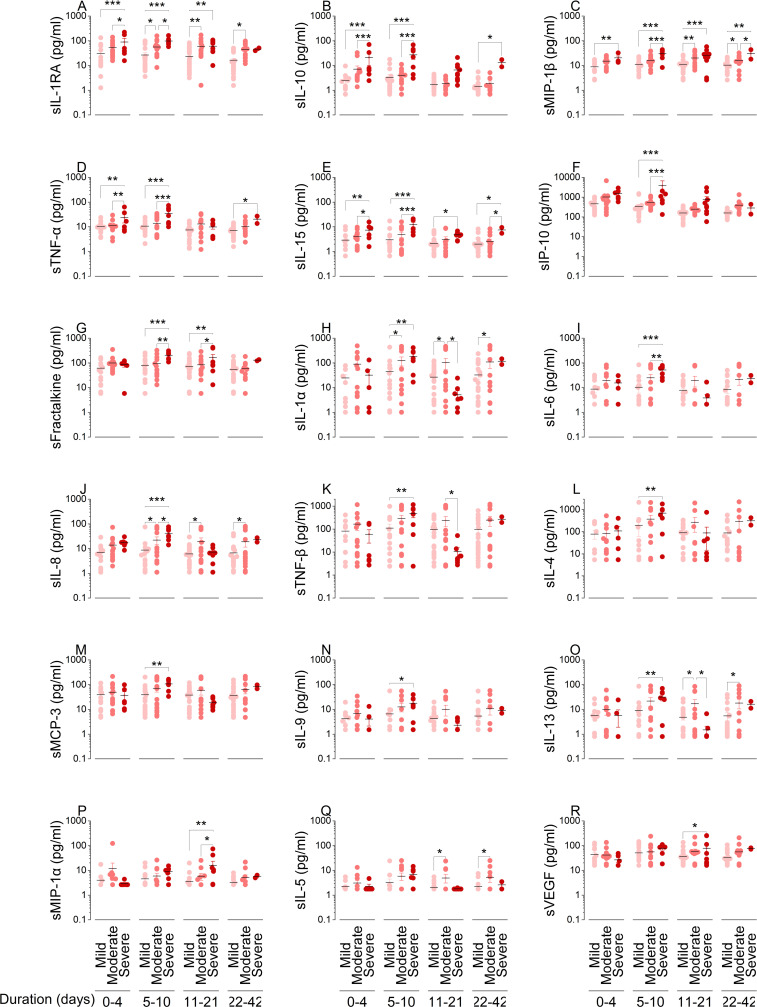
Systemic cytokine dynamics over time impact disease progression. Immune factors were quantified on the Luminex xMAP system with a MILLIPLEX MAP human cytokine/chemokine immunoassay (**A–R**). Systemic immune factors were quantified in 68 subjects from mild (*n* = 26), moderate (*n* = 26), and severe (*n* = 16) groups. Each circle represents a subject and their classification by COVID-19 severity outcome of mild (pink), moderate (salmon), or severe (maroon). A total of 169 plasma samples were quantified longitudinally (*n* = 67, 31, 35, and 36 samples from study days 1, 6, 14, and 28, respectively). Study day was converted to time from positive diagnosis, and samples were analyzed for early response (0–4 days), adaptive response (5–10 days), resolution (11–21 days), and convalescence (>21 days) phase. Changes in cytokines by severity and weeks were compared by two-way ANOVA with Tukey’s HSD test (*P < 0.05; **P < 0.01; and ***P < 0.001).

Distinct from the mucosa, no systemic immune factors were elevated in the mild group compared to the moderate and severe groups. During the early response phase, as severity outcomes worsened, there was a significant increase in anti-inflammatory IL-1RA and IL-10 (Fig. 4A and B), chemoattractants MIP-1β (Fig. 4C), and pro-inflammatory factors TNF-α and IL-15 (Fig. 4D and E). During the adaptive response phase, systemic IL-1RA, IL-10, MIP-1β, TNF-α, IL-15, IP-10, fractalkine, IL-1α, IL-6, IL-8, TNF-β, IL-4, MCP-3, IL-9, and IL-13 were significantly increased as severity outcomes worsened (Fig. 4A through O). Systemic IL-1RA, IL-10, MIP-1β, TNF-α, IL-15, fractalkine, MIP-1α, and VEGF were elevated during the resolution or convalescence, or in both phases, as severity outcomes worsened (Fig. 4A through E, G, P and R). Systemic IL-1α, IL-8, IL-13, and IL-5 significantly increased in moderate cases compared to mild cases after adaptive response phase (Fig. 4H, J, O and Q). In contrast to the mucosa, peripheral cytokines targeting T cells, DC, and NK cells progressively increased from mild to moderate to severe cases during the early response period (Fig. S4B). Cytokine levels targeting these cells were twofold higher in severe cases compared to mild cases, with a 3.2-fold increase in systemic IP-10. This was the opposite of NRF. In the early response phase, the mean total cytokine magnitudes were 2,709, 3,502, and 4,132 pg/mL for mild, moderate, and severe disease progression groups, respectively (Fig. S4B).

### Unraveling the coordination of mucosal and systemic responses to novel viruses

**Fig 5:**
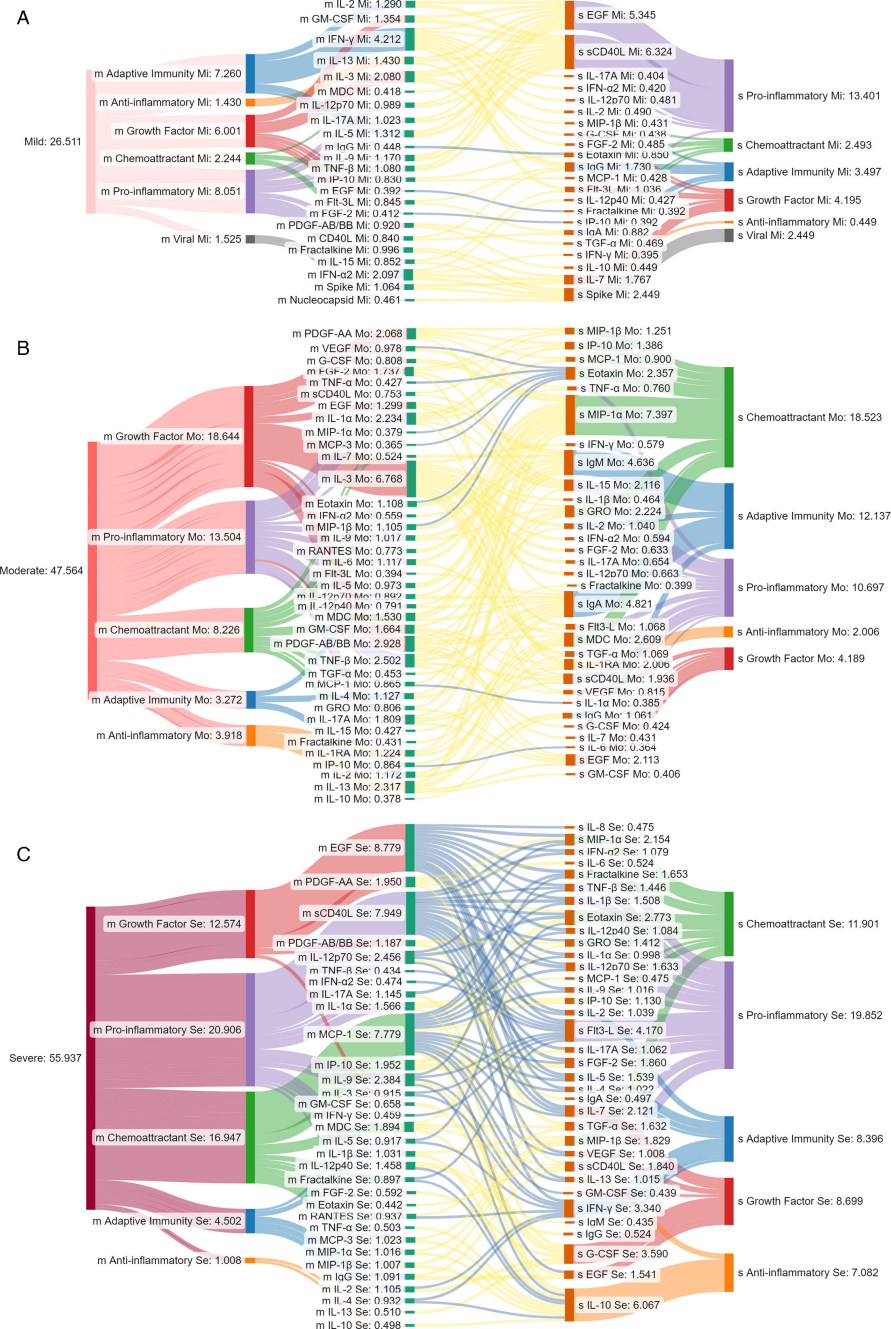
Correlation between mucosal and systemic cytokine responses by COVID-19 severity outcomes. The linear correlation between mucosal and systemic cytokines within each severity group was measured in the acute phase (≤7 days from diagnosis). The correlation between acute-phase mucosal and systemic compartments was mapped for mild (**A**), moderate (**B**), and severe (**C**) cases. The absolute value of the Pearson correlation coefficient for each immune factor with significant correlations between NRF and plasma, | *r* | > 0.35 and *P* < 0.05, was input into the model. Mucosal (M) immune factors are on the left, connected to systemic (S) compartments on the right by chords colored yellow for positive and blue for negative correlations. The node heights are proportional to the total | *r* | values indicated on the diagram. Immune factors were classified by their major function: growth factors (red), anti-inflammatory cytokines (orange), adaptive immunity cytokines (blue), pro-inflammatory cytokines (purple), and chemokines (green). Cumulative *r* values for each functional group correspond to the starting and end node thickness (**A, B, and C**).

We assessed viral and immune dynamics in both the compartments using Pearson’s correlation analysis across all participants. Significant positive and negative correlations during the acute phase of infection were traced between the mucosa and systemic compartments (Fig. 5 depicted in yellow and blue, respectively). Interestingly, only mild cases showed mucosal and systemic viral correlations (Fig. 5A, top black). Many mucosal and systemic immune factors were significantly positively correlated in individuals with mild illness, including mucosal adaptive, pro-inflammatory, anti-inflammatory, chemoattractant, viral, and growth factors, which correlated positively to systemic cytokines, except mucosal IgG, EGF, and FGF-2, which correlated negatively with systemic eotaxin, fractalkine, and IP-10, respectively (Fig. 5A). Patients with moderate illness showed increased positive and negative correlations (mVEGF, mTNF-α, mMIP-1α, mMCP-3, mEotaxin with sEotaxin, mMCP-1 with sIL-1α, and mIP-10 with sIL-6 negatively correlated) between both the compartments compared to mild illness (Fig. 5B). Although significant positive correlations were detected, we observed a dramatic increase in significant negative correlation between mucosal and systemic cytokines in severe COVID-19 cases (Fig. 5C). In the recovery phase, mild and moderate cases lacked significant negative correlations between mucosal and systemic cytokines (*r <* 0.5). In contrast, the significant negative correlations between mucosal and systemic compartments increased from the acute to recovery phases in severe cases (Fig. S5A and B).

### Prognostic indicators: IFN-α2, IP-10, IL-1RA, IL-6, eotaxin, IgA, and IgM

**Fig 6:**
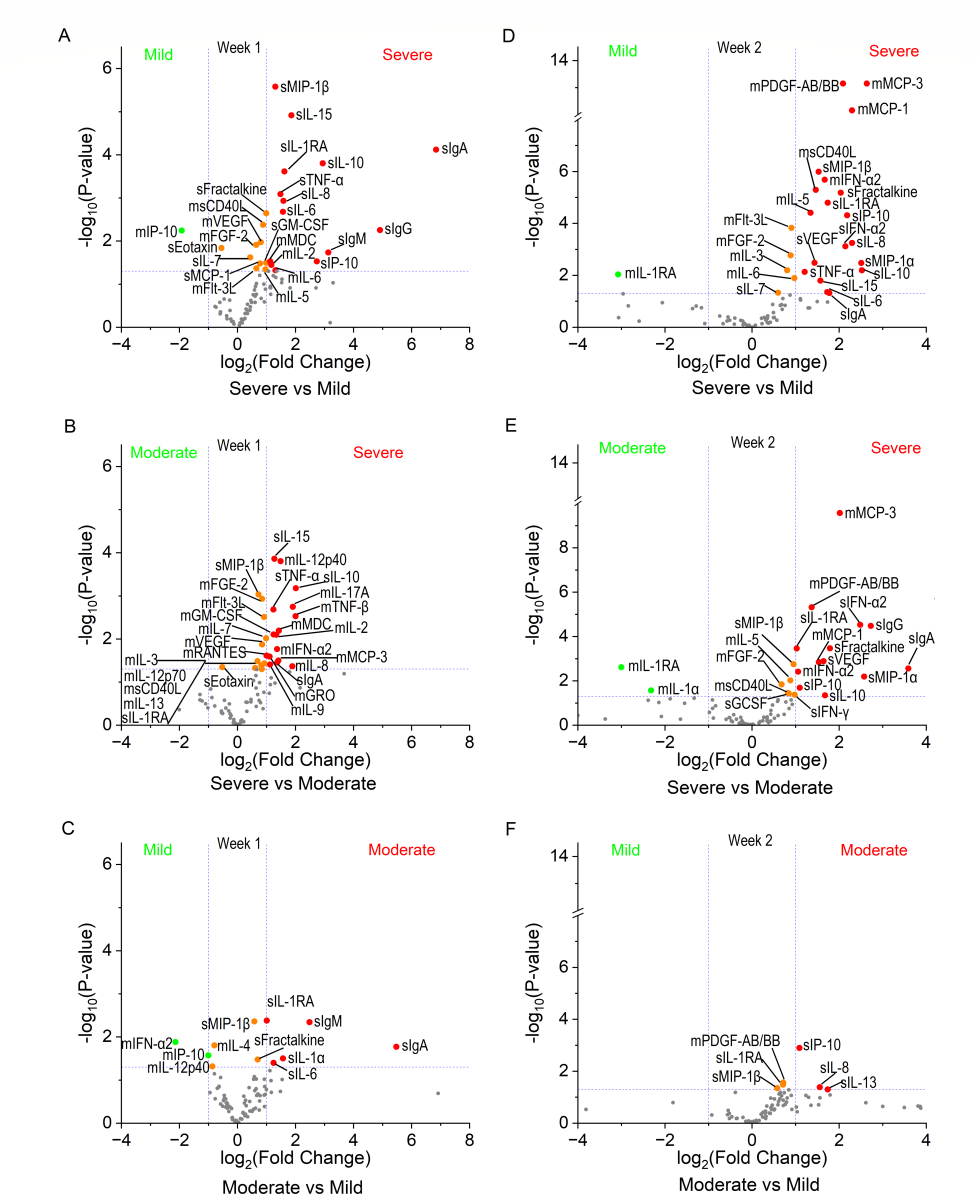
Significant differences in mucosal and systemic immune dynamics associated with varying degrees of COVID-19 disease progression. Quantitative data for NRF and plasma from 41 cytokines, chemokines, and growth factors and N- and S-specific IgA, IgM, and IgG were analyzed. Study day was converted to time from positive diagnosis ≤7 days (**A–C**) and 8–14 days (**D–F**). Significant fold changes between patients who developed severe COVID-19 were determined compared to those with mild illness (top panel) and those with moderate COVID-19 (middle panel). Significant fold changes in immune factors between non-hospitalized participants with asymptomatic or mild symptoms (mild) were determined compared to those with moderate illness (bottom panel). Differential expression analysis was performed using XLSTAT analysis set to parametric test type and Tukey (HSD). Volcano plots were graphed using OriginPro. The X-axis represents log2 fold change of protein with dashed lines intersecting at the twofold cutoff point. The Y-axis represents -log10 *P* value with dashed lines intersecting at the cutoff for significance (*P* < 0.05). Proteins that significantly increased ≥2-fold are colored red, significantly decreased ≥2-fold are colored green, and significantly different proteins with fold changes between −2 to +2 are colored orange. Mucosal (M) and systemic (S) compartments are indicated before protein symbols.

To identify potential prognostic biomarkers among naïve individuals encountering a novel virus for the first time, we performed systems analysis combining the mucosal and plasma data collected during week 1, week 2, week 3, and week 4+ from diagnosis corresponding to the early acute, late acute, recovery, and convalescent phases of infection, respectively. Within a week of diagnosis, individuals who developed asymptomatic or mild COVID-19 could be distinguished by significantly increased mucosal IP-10 and decreased systemic IL-1RA, MIP-1β, IL-6, fractalkine, IgA, and IgM within one week of positive diagnostic (Fig. 6A and C). In this early acute phase, patients who progressed to severe COVID-19 were distinguishable from those who developed mild and moderate illness by their significantly higher mucosal CD40L, FGF-2, Flt-3L, IL-2, MDC, and VEGF, as well as systemic TNF-α, IL-10, IL-15, MIP-1β, IL-1RA, and IgA, and decreased systemic eotaxin (Fig. 6A and B). In week 2, mild cases displayed significantly less mucosal PDGF-AB/BB and systemic IP-10, IL-8, IL-1RA, and MIP-1β compared to those with severe and moderate illness (Fig. 6D and F). At this time, severe cases produced significantly more mucosal IFN-α2, sCD40L, PDGF-AA/BB, MCP-1, MCP-3, IL-5, and FGF-2 and peripheral IFN-α2, IL-1RA, IP-10, IL-10, fractalkine, MIP-1α, MIP-1β, VEGF, and IgA (Fig. 6D and E). Notably, in week 2, individuals with mild to moderate symptoms produced significantly more mucosal IL-1RA than severe cases (Fig. 6D and E). Remarkably, patients with severe COVID-19 maintained significantly higher systemic IP-10, IL-1RA, IL-10, IL-15, and MIP-1β levels and mucosal IL-1RA, IL-12p40, and fractalkine through recovery and convalescence (Fig. S6A and B).

### Distinct immune responses in vulnerable understudied populations

To better understand the immune response to novel respiratory viruses in vulnerable populations, subjects were first grouped by outpatient or hospitalized based on COVID-19 severity scores. After that, subjects were grouped by age, sex, or race. The subjects were categorized by age into children (aged 8 months to 19 years) and adults (20–65 years), respectively. When outpatient children were compared to outpatient adults, children produced significantly more mucosal IL-2p70 and low levels of mucosal EGF-2 (Fig. S7A). Surprisingly, hospitalized children produced elevated levels of systemic cytokines and reduced levels of mucosal cytokines IFN-Ƴ, sCD40L, fractalkine, and Flt.3L compared to hospitalized adults (Fig. S7B).

Subjects were grouped by sex into female or male based on CRF data. Similarly, we compared outpatient females and males with COVID-19 illness. Outpatient females produced increased levels of systemic IgM with mucosal eotaxin and IgA and reduced levels of mucosal IL-1α, IL-17A, and IFN-α2 compared to outpatient males (Fig. S7A). Hospitalized females produced elevated levels of mucosal cytokines and reduced levels of systemic cytokines GM-CSF, IL-2, and IL-12p40 compared to hospitalized males (Fig. S7B). Interestingly, females showed elevated levels of mucosal eotaxin compared to males for both outpatient and hospitalization (Fig. S7A and B),

Based on CRF data, subjects were grouped by race as African Americans (AA) or European Americans (EA). AA had significantly higher severity outcome scores (mean difference = 3.453, *P* < 0.0001). Outpatient AA had significantly higher systemic cytokines, with increased mucosal IL-1α and reduced levels of mucosal RANTES, MDC, TGF-α, VEGF, and GM-CSF and systemic MCP-1 compared to outpatient EA (Fig. S7A). When hospitalized AA were compared to hospitalized EA, AA showed high levels of mucosal sCD40L, MCP-1, MCP-3, IL-1α, GM-CSF, IL-10, MDC, and EGF with systemic IgA and reduced levels of systemic EGF, GM-CSF, IL-13, sCD40L, and MCP-3 with mucosal IgA and IgG (Fig. S7B). Interestingly, AA produced elevated levels of mucosal IL-1α compared to their counterparts for both outpatient and hospitalization (Fig. S7A and B).

## DISCUSSION

COVID-19 remains a public health threat especially in the winter when hospitalizations are highest for older adults and children. Peripheral vaccination has improved outcomes but fails to elicit lasting protection against SARS-CoV-2 and durable mucosal immunity. Mucosal responses are the first line of defense against respiratory viruses and can limit their spread. In this study, we investigated the dynamics and function of mucosal and systemic immune responses to SARS-CoV-2. We used a cohort representative of the general unvaccinated, naïve population and examined the role of synchronicity between these compartments in disparate clinical outcomes. We verified that the subjects were infected by the ancestral B.1 lineages and were at the same stage of infection based on IgM seropositivity (25).

Systemic and mucosal humoral immune responses are crucial for combating respiratory viral infections and may be especially important against newly emerging viral pathogens. We found that individuals with mild COVID-19 gradually increased mucosal IgG production. Disease severity was independent of early viral load and mucosal IgA levels, indicating they do not account for desperate outcomes. We also found peripheral S levels, rate of antibody class switching, and early antibody levels significantly increased with illness severity. Antibody affinity has been reportedly lower in severe COVID-19 cases (26). We found that individuals with mild symptoms slowly increased antibody production over time, yielding significantly higher neutralization potency. Their systemic IgG levels significantly and positively correlated with neutralization, suggesting enhancement by IgG. We and others detected very high levels of systemic IgA in severe COVID-19 cases (26). IgA interference, first identified in humans following HIV vaccination (27), could negatively impact COVID-19 outcomes. Our data showed that early systemic IgA in mild cases was below the limit of detection and significantly higher in moderate and severe patients, suggesting that IgA interference may contribute to COVID-19 disease progression. We observed equivalent levels of mucosal IgA and IgG, as well as systemic IgG, concomitant with undetectable IgA and IgG in mild cases during acute infection. These initial humoral responses, which are classically considered to be more specific, did not improve clinical COVID-19 outcomes. Additionally, the higher neutralization potency we observed in mild cases is likely to improve their recall responses and enhance protection against subsequent infections. Overall, among previously naïve individuals, more gradual and synchronous mucosal and systemic humoral responses were associated with mild illness, whereas premature excessive, uncoordinated, and less potent humoral responses were observed as COVID-19 severity escalated. Our results shed new light on the discrepancy between individuals who have restrained humoral responses to novel viruses and those who initiate early vigorous peripheral IgA production and develop more severe symptoms. They also support the notion that mucosal vaccines could serve as a valuable option for future pandemics and may offer more sustained and potent protection than current COVID-19 booster strategies that increase systemic IgA.

Neutralizing antibodies alone are insufficient for viral clearance. We found that naïve individuals with mild COVID-19 cleared the virus in week 1 and produced significantly more mucosal IFN-α and IP-10 within four days of diagnosis, indicating robust very early antiviral responses. This is consistent with other COVID-19 studies associating severe disease with impaired epithelial interferon responses, fewer local IFN-α-producing plasmacytoid dendritic cells, and mucosal IP-10 levels that correlated with interferon responses (28–30). Mild cases were marked by the immediate production of IFN-α and IP-10 at the site of infection, enhancing the recruitment of cells expressing the cognate CXCR3 receptor, such as T and NK cells. This can expedite viral clearance, the production of germinal center-derived high-affinity antibodies, and the control of systemic immune responses. In week 2, we observed that mild and moderate cases had significantly higher mucosal anti-inflammatory IL-1RA compared to those with severe illness, suggesting that IL-1RA mediated the return of local immune responses to baseline. Systemic IFN-I responses were reportedly diminished or absent in hospitalized patients with COVID-19 (31). However, we found delayed mucosal and systemic IFN responses in patients with severe COVID-19, which could allow for deeper virus penetration in the respiratory tract early in the infection. Our analysis revealed that naïve individuals with synchronized mucosal and systemic cytokine responses in the acute phase develop mild illness, whereas those with uncoupled local and peripheral early responses progress to more severe illness and fail to adequately resolve immune responses to novel viruses in the recovery phase. Overall, these data strongly support a role for early mucosal IFN-α and IP-10 in antiviral protection and, in conjunction with subsequent IL-1RA production, in limiting disease severity in the naïve population.

While the number of participants enrolled in first wave infections limited the statistical power of our subgroup analyses, our data suggest novel differences in COVID-19 cytokine response and outcomes in race, sex, and age subgroups. Children, females, and AA may have unique immune responses to SARS-CoV-2 that could benefit from specific clinical interventions. The high levels of IL-6 and IL-8 observed here indicate that tocilizumab and siltuximab may be particularly effective for treating hospitalized pediatric and female patients with COVID-19. Male sex and low levels of eotaxin have been reported in severe COVID-19 illness (32). Our results show that high levels of eotaxin in females could be protective against poor COVID-19 outcomes. IL-1 receptor antagonism via anakinra has improved clinical outcomes for severe COVID-19 (33). We found that AA and hospitalized children had elevated IL-1, suggesting that anakinra may be especially effective in these populations. Our analysis of the entire cohort suggests that IFN-α2, IP-10, IL-1RA, IL-6, eotaxin, IgA, and IgM are prognostic markers that can be monitored in mucosal fluids and blood at the time of diagnosis or shortly after to forecast disease progression and aid in treatment plans following infection with a novel respiratory virus. Importantly, these findings came from a cohort with equal representation across outcomes, sex, age, and race subgroups, which increases the generalizability of this study. To our knowledge, this is the most diverse cohort to have a COVID-19 mucosal immunity assessment and the first reported comparison of mucosal and peripheral immune kinetics extending into convalescent infection.

This study has several limitations. First, although our cohort was racially diverse and balanced by sex and age, the overrepresentation of severe cases among African American participants and mild cases among European Americans limits the generalizability of race-stratified subgroup analyses. Second, we did not assess prior exposure to seasonal human coronaviruses, which could influence immune priming. While pre-existing humoral and cellular immunity to SARS-CoV-2 has been reported in unexposed individuals, the literature remains mixed on whether such responses modulate disease severity or confer protection. Importantly, the current consensus suggests that pre-existing cross-reactive T-cell responses may offer modest benefits (34, 35), while cross-reactive antibodies are less likely to be protective (34, 36, 37). Third, participants were enrolled between July 2020 and March 2021, when cumulative case counts in the area remained low (between 0.93% and 10% of the population, respectively), and epidemiologic evidence indicates that prior infection conferred strong protection against reinfection for at least 6 to 12 months. Given this low prevalence and the durability of post-infection immunity during the pre-Omicron period, it is highly likely—though not certain—that participants were experiencing a primary SARS-CoV-2 infection. Consistent with this, nearly all were IgM-positive by week 2, supporting recent infection and appropriate cohort classification. Fourth, we standardized sampling time points using the date of first positive SARS-CoV-2 PCR test rather than symptom onset due to variability and underreporting in self-reported symptoms—especially among Black participants. This decision was supported by serological validation and enabled consistent temporal alignment across the cohort. Finally, this observational study establishes associations—but not causality—between immune parameters (e.g., mucosal IFN-α, IP-10, IL-1RA) and clinical outcomes in SARS-CoV-2–naïve individuals. Nonetheless, our findings are consistent with emerging reports in vaccinated and breakthrough cases that identified these cytokines as systemic markers of vaccine responsiveness or protection (38–40). Thus, although our findings reflect immune responses in SARS-CoV-2–naïve individuals, particularly elevated mucosal IFN-α and IP-10 followed by IL-1RA, they may have broader translational relevance.

In conclusion, our data provide strong evidence of the vital role of early mucosal responses in controlling disease progression following infection with a novel respiratory virus, such as SARS-CoV-2, and highlight the potential power of targeting mucosal immunity in vaccination efforts and monitoring mucosal responses in the naïve population. Individuals with high mucosal IFN-α, IP-10, and IL-1RA production quickly synchronized local and systemic immune responses, whereas those lacking these protective mucosal responses had poor or dysregulated immune responses with delayed resolution that were associated with more severe illness. We began collecting nasopharyngeal rinses and blood in 2020 from participants with a broad range of COVID-19 severity, a cohort that will be impossible to replicate in the future due to acquired immunity. This unique study revealed previously unknown aspects of mucosal responses to SARS-CoV-2 associated with disease progression in the naïve population. These data and insights may prove useful for developing next-generation COVID-19 vaccines and prognostic indicators when the next novel respiratory virus emerges.

## MATERIALS AND METHODS

Additional details are provided in the online data supplement.

### Study participants

Participants with COVID-like illnesses were recruited from Le Bonheur Children’s Hospital, Methodist University Hospital, and outpatient testing sites in Memphis, Tennessee. Pregnant women and those who could not provide informed consent were excluded. Participants with clinical laboratory polymerase chain reaction or antigen positive results within 72 hours of enrollment were included. Two severe subjects died during the study. Two seniors were partially vaccinated against SARS-CoV-2. Electronic medical record data were collected using a standardized data record form.

### Study procedures

Mid-turbinate swabs (MT-swabs) were collected and placed in viral transport media on ice. Then, 0.1% saline was flushed through the nares to collect nasopharyngeal rinse fluid (NRF), followed by the addition of ice-cold BEGM. The sample was centrifuged, cells were removed, and protease inhibitor cocktail was added. Blood was drawn into vacutainer cell preparation tubes and immediately processed following the manufacturer’s guidelines. Aliquots were stored at −80°C.

### Primary outcomes

COVID-19 outcome was assigned based on retrospective chart review at least 28 days from diagnosis. A modified World Health Organization (WHO) COVID-19 case definition was used to score severity outcome (41, 42), based on hospitalization and symptoms (Table 1). Participants with asymptomatic infections were classified as mild. All outpatients were classified as mild, and hospitalized participants were designated as moderate or severe.

To standardize temporal analyses, the date of the first positive SARS-CoV-2 PCR test was designated as day 0 for all participants. Time from symptom onset to diagnosis varied across individuals and was not used as an anchor due to reliance on self-report. To confirm that PCR-based day 0 reflected early-stage infection, SARS-CoV-2-specific IgM levels were measured across time points. The majority of participants were IgM-positive by week 2, consistent with expected early seroconversion (Fig. S2).

### SARS-CoV-2 RNA RT-qPCR and sequencing

RNA was isolated from swabs and subjected to reverse transcription real-time quantitative polymerase chain reaction (RT-qPCR). Primers targeted either ORF1b-nsp14 or Spike (43). Average cycle threshold (Ct) value was determined with a negative threshold of 40. PCR-positive swabs were sequenced following the manufacturer’s protocols. RNA was transcribed to single-stranded cDNA using random hexamers. SARS-CoV-2 sequence libraries were prepared and sequenced using paired-end 2 × 150 bp reads with the MiSeq Reagent Kit v2 for 300 cycles (Illumina, #MS-102-2002). The sequenced libraries were assembled, and SARS-CoV-2 lineages were determined as previously described (44).

### Antigen and antibody quantification

S, N, and antibodies were quantified by ELISA. Undiluted NRF and plasma were quantified and analyzed per manufacturer recommendations. Protein concentrations were extrapolated from standard curves.

### Neutralization assays

Plasma neutralization of spike-containing pseudovirus was performed as previously described (45). Antibody neutralization potency was calculated as a ratio of neutralization titer 50 (NT_50_) to the sum of plasma antibodies (46). Neutralizing antibody activity was quantified using the BioPlex Pro Human SARS-CoV-2 Neutralization Antibody 2-Plex Panel (BioRad #12016848), which operates via a bead-based competitive assay measuring inhibition of ACE2 binding to RBD and S1 antigens. Antibody concentration and percent inhibition were calculated based on MFI values relative to negative controls and standard curves.

### Immune factor quantification

Forty-one human immune factors were quantified on a Luminex 200 instrument according to the manufacturer’s instructions. The absolute quantity of immune factors was reported as pg/mL of plasma and pg/mg protein of concentrated NRF.

### Statistical analysis

GraphPad Prism software was used for basic statistical analysis: Log-rank Mantel-Cox test, one-way ANOVA with Fisher’s least significant difference (LSD) procedure and posttest for linear trends, and two-way ANOVA with Tukey’s honestly significant difference (HSD). The XLSTAT package was used for Pearson correlations and expression analysis. Post hoc power analyses were conducted using standard statistical methods to assess the sensitivity of our sample sizes for detecting group differences or correlations across various experiments summarized in the figures. The power analyses summarized below were performed using the statistical software G*Power 3.1 (47).

#### Humoral immune analyses

Group comparisons of N- and S-specific Ab levels in mucosal (NRF) and systemic (plasma) samples were analyzed using one-way ANOVA with Fisher’s LSD post hoc test. Sample sizes per severity group were as follows: mild (*n* = 102 NRF, 86 plasma), moderate (*n* = 77 NRF, 62 plasma), and severe (*n* = 23 NRF and plasma). At α = 0.05, these group sizes provide ≥80% power to detect moderate-to-large differences in mean antibody levels (Cohen’s *f* ≥ 0.4). The severe group had ≥70% power for large effects (*d* ≥ 0.8). Correlation analyses using 130 paired NRF-plasma samples had >90% power to detect moderate associations (*r* ≥ 0.3) at α = 0.05.

#### Cytokine analyses

Cytokine levels were compared across severity groups at defined time windows using two-way ANOVA with Tukey’s HSD correction. Mucosal cytokines were quantified in 190 nasopharyngeal rinse fluid (NRF) samples from mild (*n* = 59), moderate (*n* = 44), and severe (*n* = 43) cases. Systemic cytokines were quantified in 169 plasma samples from mild (*n* = 67), moderate (*n* = 31), and severe (*n* = 35) cases. Based on these sample sizes and α = 0.05, the analyses had ≥80% power to detect moderate-to-large between-group differences in cytokine levels (Cohen’s f ≥ 0.4) at early (0–4 days) and adaptive (5–10 days) time points. To assess early associations between mucosal and systemic cytokines within severity groups, we analyzed NRF and plasma samples collected during the acute phase (≤7 days from diagnosis): mild (*n* = 24 NRF, 24 plasma), moderate (*n* = 25 NRF, 21 plasma), and severe (*n* = 10 NRF, 12 plasma). Power analysis (α = 0.05, two-tailed) indicates ≥80% power to detect correlations (*r* ≥ 0.4) in mild and moderate groups and (*r* ≥ 0.62) in the severe group.

#### Between-group immune comparisons at defined time windows

Between-group comparisons of mucosal and systemic immune responses—including cytokines, chemokines, growth factors, and N/S-specific IgA, IgM, and IgG antibodies—were conducted using parametric statistical tests with Tukey’s HSD post hoc correction at two defined time windows (week 1: ≤7 days post-diagnosis; week 2: 8–14 days). Sample sizes were mild (*n* = 161), moderate (*n* = 121), and severe (*n* = 66). Based on these group sizes and α = 0.05, the estimated statistical power for detecting differences in immune markers was as follows: mild vs. moderate: ≥95% power for moderate effects (Cohen’s *d* ≥ 0.5), ≥99% for large effects (d ≥ 0.8); mild vs. severe: ≥80% power for moderate effects, ≥98% for large effects; and moderate vs. severe: ≥72% power for moderate effects, ≥95% for large effects.

## SUPPLEMENTAL MATERIAL

Fig. S1 mbio.01491-25-s0001.tif SARS-CoV-2 viral and infection stage characterization.

Fig. S2 mbio.01491-25-s0002.tif Mucosal and systemic S and N-specific antibody production significantly altered by disease progression and week from diagnosis

Fig. S3 mbio.01491-25-s0003.tif Mucosal and systemic cytokine expression.

Fig. S4 mbio.01491-25-s0004.tif Enhanced mucosal with diminished systemic cytokine targeting of dendritic, NK, and T cells is associated with milder COVID-19 progression.

Fig. S5 mbio.01491-25-s0005.tif Correlation plots for mucosal and systemic immune factors responses during acute and recovery phases.

Fig. S6 mbio.01491-25-s0006.tiff Significant differences in mucosal and systemic immune factor dynamics are associated with varying degrees of COVID-19 disease severity in recovery.

Fig. S7 mbio.01491-25-s0007.tif COVID-19 induces differential expression of immune factors in children and African Americans.

Supplemental material mbio.01491-25-s0008.docx Supplemental text and figure legends.

ASM does not own the copyrights to Supplemental Material that may be linked to, or accessed through, an article. The authors have granted ASM a non-exclusive, world-wide license to publish the Supplemental Material files. Please contact the corresponding author directly for reuse.
