# Supplementary figures and images for: Early mucosal IFN-α, IP-10, and IL-1RA and synchronized mucosal and systemic immune responses mediate COVID-19 disease progression
Source: mBio. 2025 Nov 28;17(1):e01491-25. doi: 10.1128/mbio.01491-25 (PMC12802262; doi:10.1128/mbio.01491-25)

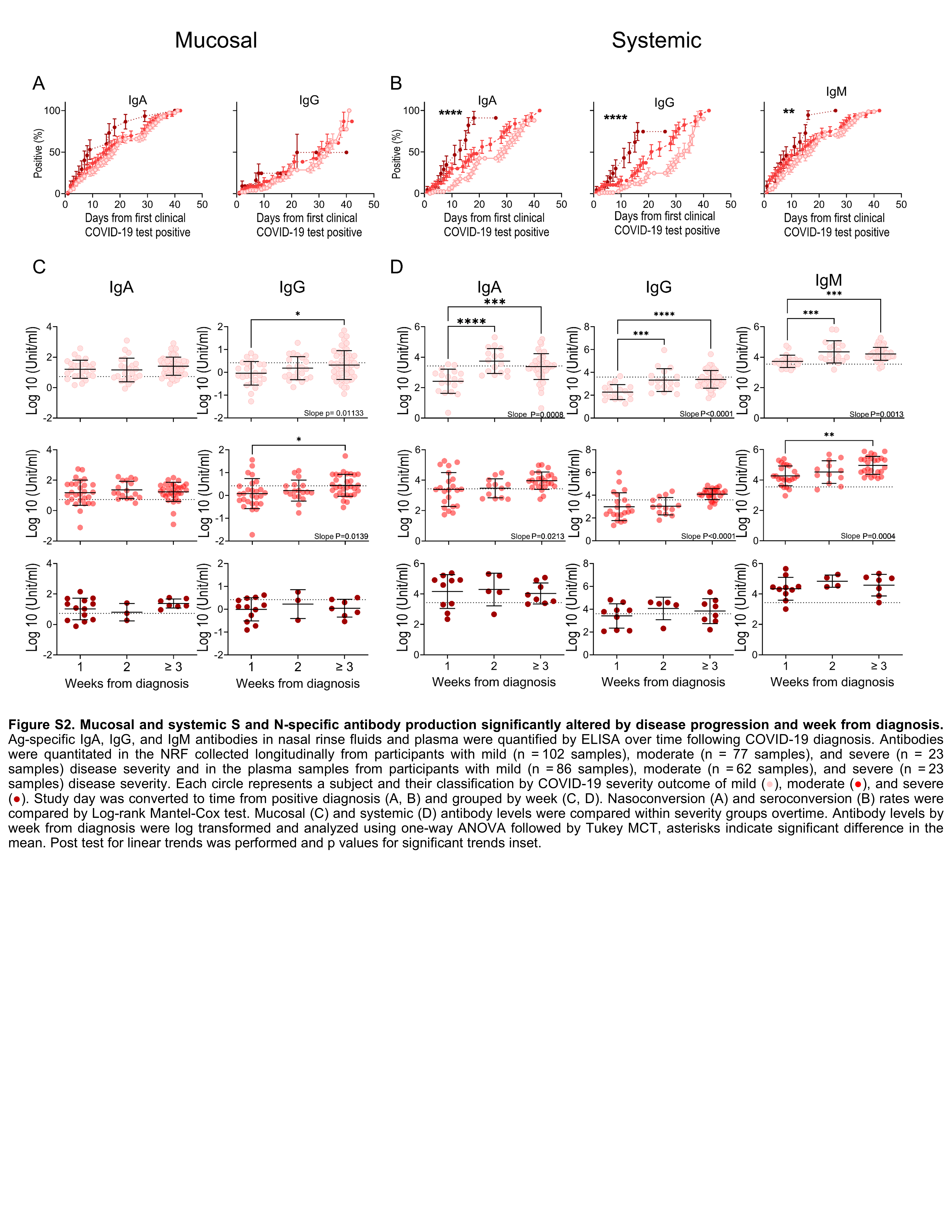

Supplement: Fig. S2 — Mucosal and systemic S and N-specific antibody production significantly altered by disease progression and week from diagnosis [file mbio.01491-25-s0002.tif]

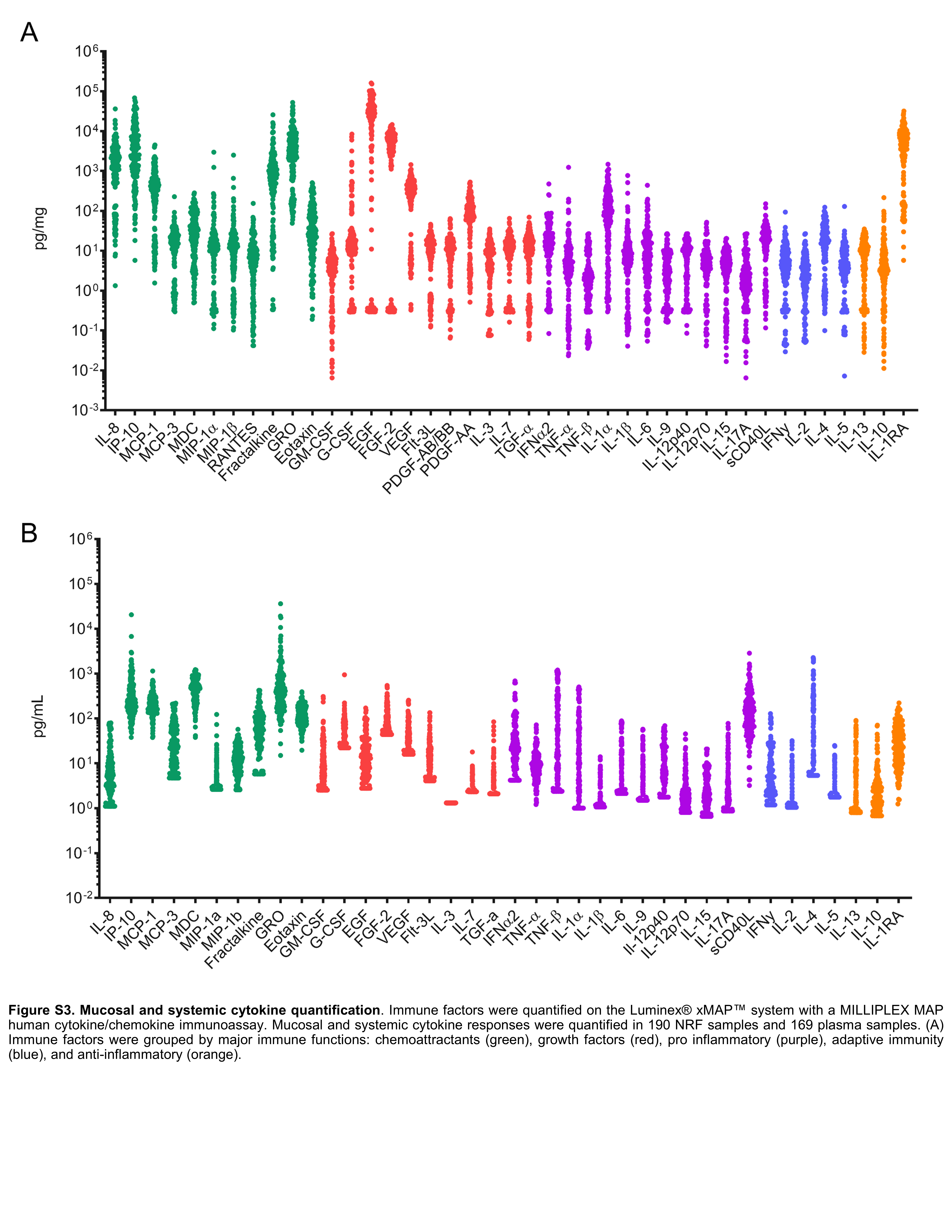

Supplement: Fig. S3 — Mucosal and systemic cytokine expression. [file mbio.01491-25-s0003.tif]

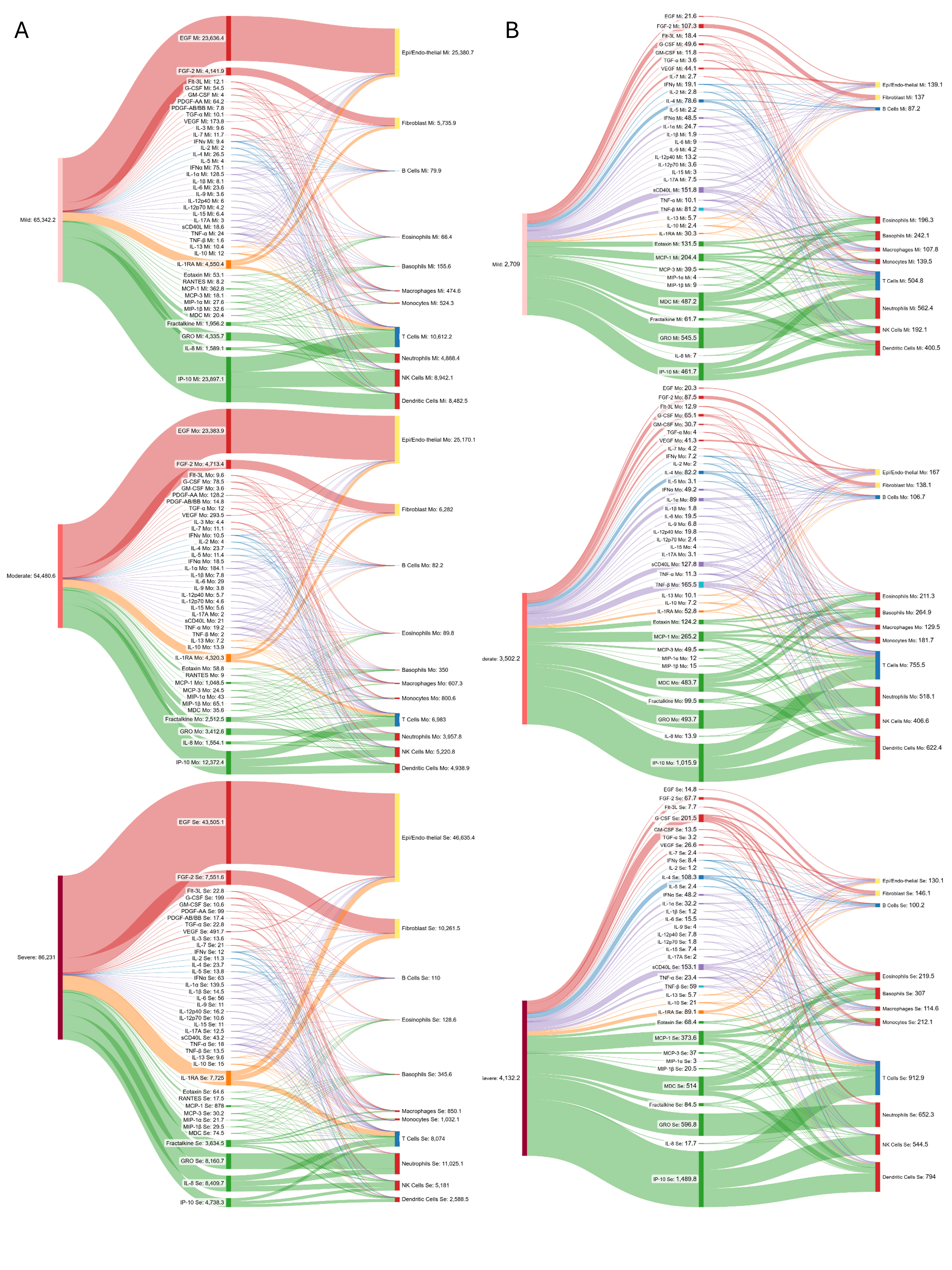

Supplement: Fig. S4 — Enhanced mucosal with diminished systemic cytokine targeting of dendritic, NK, and T cells is associated with milder COVID-19 progression. [file mbio.01491-25-s0004.tif]

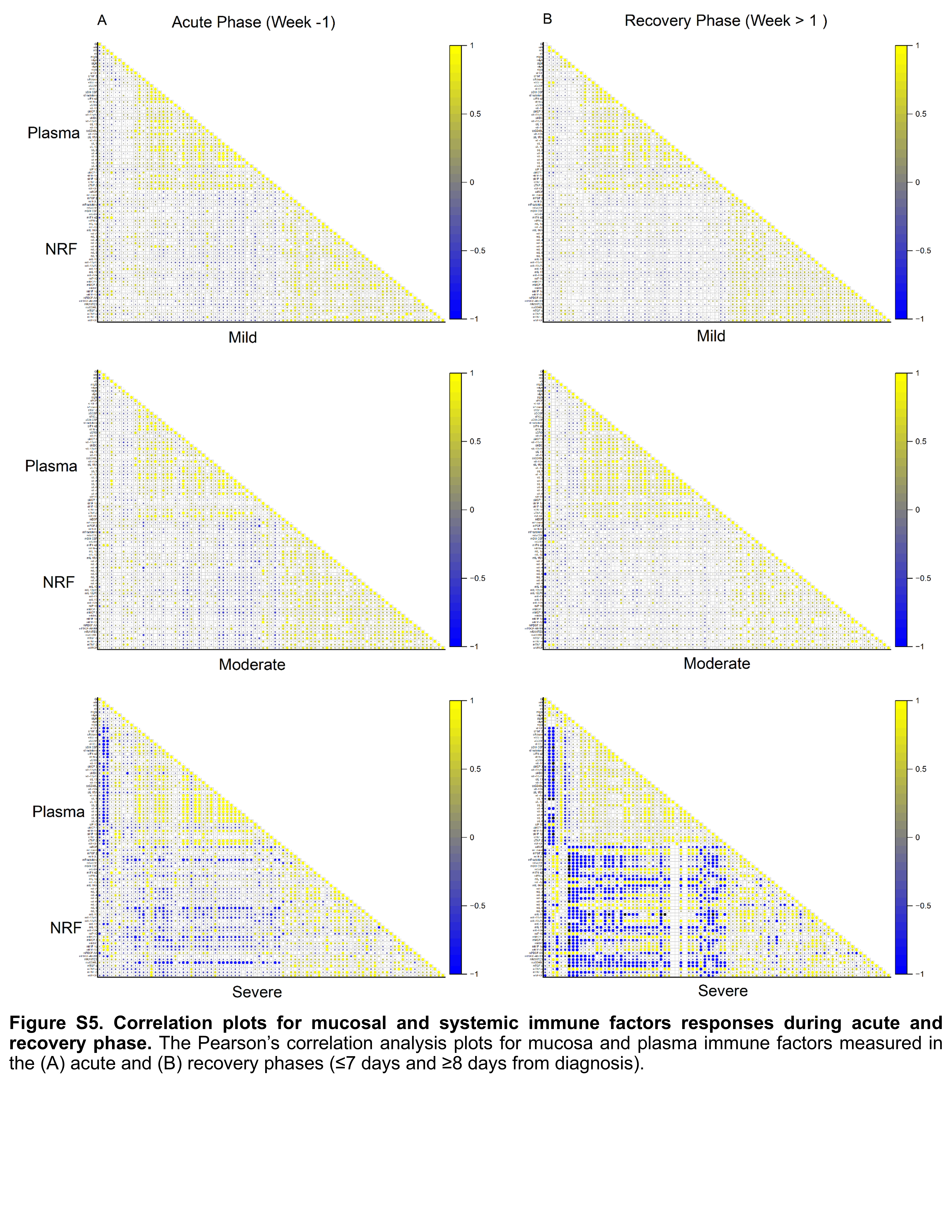

Supplement: Fig. S5 — Correlation plots for mucosal and systemic immune factors responses during acute and recovery phases. [file mbio.01491-25-s0005.tif]

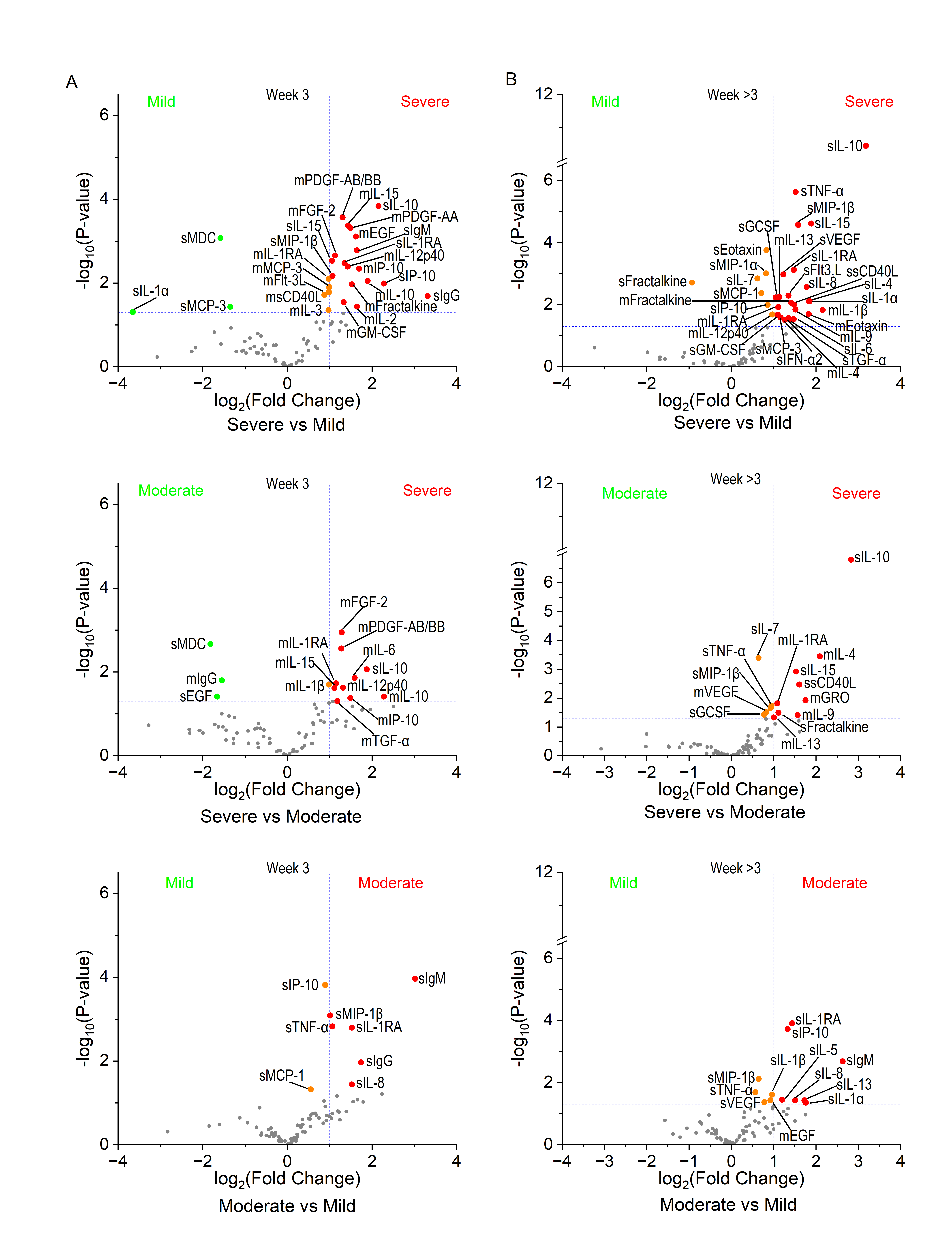

Supplement: Fig. S6 — Significant differences in mucosal and systemic immune factor dynamics are associated with varying degrees of COVID-19 disease severity in recovery. [file mbio.01491-25-s0006.tiff]

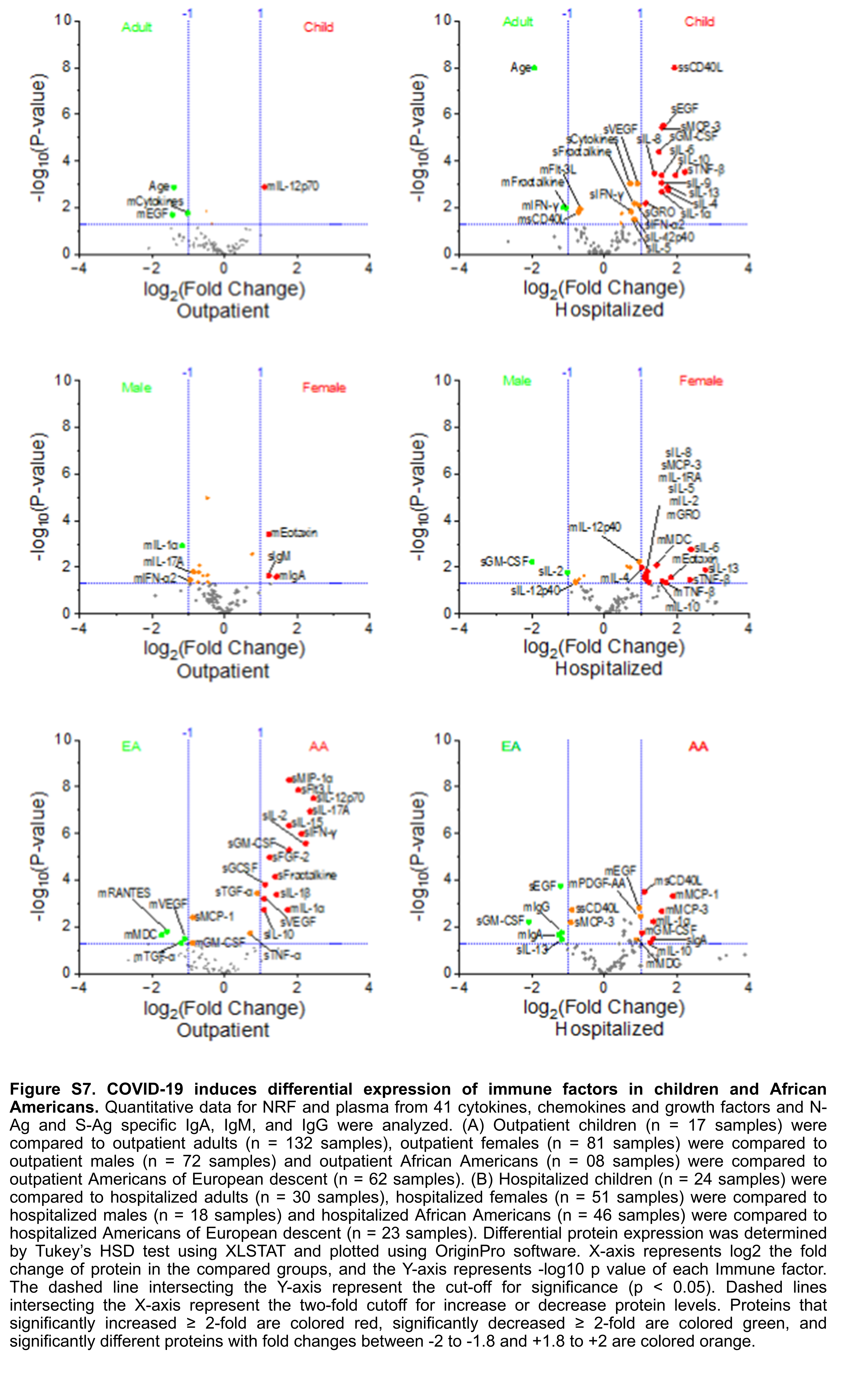

Supplement: Fig. S7 — COVID-19 induces differential expression of immune factors in children and African Americans. [file mbio.01491-25-s0007.tif]
