# Supplementary material for: Early mucosal IFN-α, IP-10, and IL-1RA and synchronized mucosal and systemic immune responses mediate COVID-19 disease progression
Source: mBio. 2025 Nov 28;17(1):e01491-25. doi: 10.1128/mbio.01491-25 (PMC12802262; doi:10.1128/mbio.01491-25)
Supplement: Supplemental material — Supplemental text and figure legends. [file mbio.01491-25-s0008.docx]

**Online Data Supplement**

Title: Mucosal IFN-α, IP-10, and IL-1RA Mediate Viral Clearance and COVID-19 Disease Progression.

Mona Agrawal,^1^ Armando S. Flores-Torres,^1^ John S. Franks,^2^ Sarah Y. Lang,^3^ Thomas P. Fabrizio,^2^ Kristin E. McNair,^4^ Laura V. Boywid,^5^ Ashley J. Blair, ^1^ Chloe N. Hundman,^4^ Nicholas D. Hysmith, ^1,4,5^ Michael A. Whitt,^3^ Rachael Keating,^6^ Paul G. Thomas,^7^ Richard J. Webby,^7^ Amanda M. Green,^1,2,4 ŧ^ and Heather S. Smallwood^1,4,5 ŧ*^

**Supplementary Methods**

**Regulatory Compliance**

The University of Tennessee Health Science Center (UTHSC) UTHSC and St. Jude Children’s Research Hospital Institutional Review Boards approved this study. Participants or their legal guardians provided written informed consent in compliance with 45 CFR 46 and The Declaration of Helsinki.

**Inclusion and exclusion criteria:** Non-English-speaking persons, pregnant women, and those who could not provide informed consent or lacked an available designated decision maker were excluded from the study. Participants visited presented to local hospitals or to the UTHSC’s community COVID-19 testing site to have had SARS-CoV-2 testing due to symptoms or known exposure to COVID-19 positive individuals were included in the study.

**Human Subjects and Specimens:** The Institutional Review Boards of The University of Tennessee Health Science Center (UTHSC) and St. Jude Children’s Research Hospital approved this study. Participants or their legal guardians provided written informed consent in compliance with 45 CFR46 and the Declaration of Helsinki regarding ethical principles for medical research. Participants were recruited from Le Bonheur Children’s Hospital, Methodist Germantown Hospital, Methodist University Hospital, and UTHSC’s outpatient community COVID-19 testing sites in Memphis, Tennessee. The study inclusion criteria required participants to have had SARS-CoV-2 testing due to symptoms or known exposure to COVID-19 positive individuals. Participants were considered COVID-19 positive if SARS-CoV-2 polymerase chain reaction (PCR) tests conducted in clinical laboratory was positive. Non-English-speaking persons, pregnant women, and those who could not provide informed consent or lacked an available designated decision maker were excluded from the study.

Demographic, epidemiological, clinical, laboratory, treatment, and outcome data were collected from electronic medical records as available. Clinical features were collected using a standardized data record form. The Severity of COVID-19 illness was assigned based on retrospective chart review, completed within 28 days from enrollment. A modified World Health Organization (WHO) COVID-19 case definition was used to assign severities of asymptomatic, mild, moderate, and severe. Standardized point values were assigned based on hospital admission, intensive care unit (ICU) admission, and symptoms detailed in (**Table 1**). Participants were classified with mild, moderate, or severe COVID-19 based on cumulative point scores (i.e., total ≤1, 2–4, or ≥5, respectively). Hospitalized participants were all designated as moderate or severe, and all participants admitted to the ICU were designated as severe. All subjects that self-identified as black or white were considered to be of African or European descent. Participants were considered to be children, adults, or seniors based on ages of ≤ 19, 20 to 65, or >65 years, respectively.

Specimens were obtained immediately after enrollment on visit day 1 and in follow-up visits on days 6, 14, and 28. Mid turbinate nasopharyngeal swabs (MT-swabs) were collected. Briefly, flocked sterile swabs were inserted approximately 1 inch into the mid-turbinate region, rotated several times against the nasal walls of each nares, and placed into viral transport media (VTM). Nasopharyngeal rinses were next collected. Briefly, both nares were flushed with 0.1% saline and collected by gravity flow or aspiration and transferred to tubes containing ice-cold bronchial epithelial cell growth medium (BEGM) and processed within 2 hours of collection. Rinses were filtered using a 40 µm cell strainer with cells pelleted and removed, then the protease inhibitor cocktail was added to nasopharyngeal rinse fluids (NRF) before they were stored at -80°C. Blood was drawn into BD vacutainer CPT™ cell preparation tubes (BD Biosciences, Franklin Lakes, NJ, USA) with sodium citrate. Blood was processed the day of collection per manufacturer guidelines and plasma stored at -80°C. On study days 14 and 28, participants completed a questionnaire, which included questions to determine estimated dates of symptom onset and relief, socio-demographic and family characteristics, and medical history.

**SARS-CoV-2 RNA RT-qPCR:** MT-swabs were transported to the lab frozen or on ice. RNA was isolated and subjected to reverse transcription real-time quantitative polymerase chain reaction (RT-qPCR) and positive samples sequenced. RNA from MT-swabs were extracted using a RNeasy mini kit (Qiagen, Venlo, Netherlands, #74106) following the manufacturer’s protocol. One-step RT-qPCR reaction was performed with ABI FAST Virus 1-step Master Mix (Applied Biosystems, Waltham, MA, USA, #4444436), SARS-Cov-2 specific primers and probes targeting either ORF1b-nsp14 or Spike (in house design in collaboration with St Jude’s Center for Applied Bioinformatics, CAB) and template RNA on an ABI 7500 FAST machine (Applied Biosystems).(E1) Reverse transcription was performed for 5 minutes at 50°C, followed by 20 seconds at 95°C. The reaction was performed with 40 cycles of 95°C for 5 seconds, followed by 60°C for 30 seconds. A cycle threshold (Ct) value for each sample was determined as the average of triplicate wells per target. Samples failing to cross the threshold by cycle 40 were marked as negative.

**SARS-CoV-2 Sequencing:** SARS-CoV-2 positive MT-swabs samples were sequenced. For sequencing, RNA was isolated from samples using either the Qiagen RNeasy Mini Kit (Qiagen, #74104) or the MagMaxTM Viral/Pathogen Nucleic Acid Isolation Kit (Applied Biosystems, #A42352) on a ThermoFisher Kingfisher Flex 96-well magnetic purification platform (Thermo Fisher Scientific, Waltham, MA, USA). RNA was transcribed to single stranded cDNA using an Invitrogen SuperScript IV first strand synthesis kit (Invitrogen, Waltham, MA, USA, #18091050) using random hexamers and following manufacturer’s recommendations with the minor modification of extending the RT incubation step from 5 minutes to 1 hour. From the cDNA, SARS-CoV-2 sequence libraries were prepared using the xGen SARS-CoV-2 Amplicon Panel (Integrated DNA Technologies [IDT], Coralville, Iowa, USA, #10009832) and following the manufacturer’s protocol. Briefly, the SARS-CoV-2 genome was amplified using a multiplexed PCR creating 345 overlapping amplicons in a single reaction. Adapters and indexes were then added to the amplicons in a second limited cycle PCR. The libraries were normalized using the Normalase enzymatic treatment. Normalized libraries were quantified using the NEBNext Library Quant kit for Illumina (New England Biolabs, Ipswich, MA, USA, #E7630L) and diluted to the required loading concentration. The libraries were sequenced by paired-end 2 x 150 using the Illumina MiSeq Reagent Kit v2 (Illumina, San Diego, CA, USA, #MS-102-2002) for 300-cycles.

The sequenced libraries were assembled, and SARS-CoV-2 lineages were determined using an in-house developed pipeline called idCOV.(E2) Briefly, the reads were trimmed for quality using Trimmomatic and primer sequences were removed using Primerclip (IDT). The reads were then assembled to the original Wuhan-Hu-1 strain sequence. Mutations were identified and confidence was assigned based on read quality and depth at each location. Once the genome was constructed, mutation markers were compared to lineage-defining mutations to determine the SARS-CoV-2 lineage of each sample. Following the idCOV lineage determinations, all lineages were confirmed through UCSC UShER: Ultrafast Sample placement on Existing tRee to compare each assembled genome with all available SARS-CoV-2 sequences.

**Antigen and Antibody Quantification:** SARS-CoV-2 S and N Ag proteins were quantified in plasma and NRF by ELISA (ELV-COVID19S1 and ELV-COVID19N, respectively; RayBiotech, Peachtree Corners, GA, USA). Antibodies (IgM, IgA, and IgG antibodies) against SARS-CoV-2 S and N proteins were quantified in plasma and NRF using ELISA kits from RayBiotech (IEQ-CoVSN-IgM, IEQ-CoVSN-IgA, and IEQ-CoVSN-IgG). Technical replicates were performed. Samples were run in duplicate per manufacturer’s protocol and analyzed per manufacturer recommendations, except that the nasal fluids were not diluted. Antigen and antibody concentrations were extrapolated from standard curves.

**SARS-CoV-2/VSV pseudotype production and neutralization assays:** VSV-DG-luciferase pseudo types displaying the full-length SARS-CoV-2 spike (Wuhan-Hu-1 strain) were generated using a plasmid encoding a codon-optimized cDNA for the SARS-CoV-2 spike as described by Whitt 2010.(E3) Plasmid expressing codon-optimized cDNA for the SARS-CoV-2 spike was generously provided to us by Dr. Florian Krammer, Icahn School of Medicine, Mount Sinai. (E4) Residual infectivity from the VSV-G pseudo typed DG-luciferase inoculum was neutralized immediately after VSV-G pseudo typed DG-luciferase virus adsorption by incubation for 30 minutes with a hybridoma culture supernatant that contained the I1 VSV G-specific monoclonal antibody.(E5) SARS-CoV-2 specific titers were determined on VeroE6-TMPRSS2 cells, which were obtained from the Japanese Collection of Research Bioresources (JCRB) (National Institutes of Biomedical Innovation, Health and Nutrition, Japan, Cat #1819).(E6)

SARS-CoV-2 neutralizing activity in sera from study participants was determined in VeroE6-TMPRSS2 cells using a standard protocol. Briefly, 2 x 103 infectious units of SARS-CoV-2/DG-luciferase pseudotype virus were mixed with increasing 2-fold dilutions of patient sera that had been complement-inactivated by incubation at 56°C for 30 minutes. The virus-sera admixtures were next incubated at 37°C for 1 hour and then added directly to VeroE6-TMPRSS2 cells in a 96-well plate that had been passaged ~18 hours prior. Luciferase activity was assayed 17 hours post-inoculation using the XTND Luc-Screen kit as per the manufacturer’s instructions (Applied Biosystems) and relative light units were read using a BioTek Synergy 2 plate reader (BioTek, Winooski, Vermont, USA). The raw data were then transformed and plotted using Prism GraphPad (GraphPad Software, La Jolla, CA, USA) and neutralizing titers were determined and compared to a positive control (sera from an individual who had severe COVID-19) and a negative control serum from an uninfected individual. Ab neutralization potency was calculated as a ratio of neutralization titer 50 (NT_50_) to the sum of plasma SARS-CoV-2 specific isotypes and grouped by severity.(E7)

**Bio-Plex Neutralization Antibody Assay:** Plasma from study participants collected on either study day 28 or 14 days after the diagnosis was quantified for the percent spike and RBD antibody inhibition using Bio-Plex Pro Human SARS-CoV-2 neutralization antibody assay kit from Bio-Rad (Bio-Rad Laboratories, Hercules, CA, USA, #12016848). Neutralization antibody assay with each sample was run in duplicate and the assay was performed according to the manufacturer’s instructions and then plate was analyzed on a Luminex 200 (Luminex Multiplexing Instrument, Merck Millipore, Burlington, MA, USA) analyzer. Data obtained were extracted using xPONENT software (IQVIA, Durham, NC, USA). Percent inhibition was calculated by comparing sample mean fluorescent intensity (MFI) results to the negative control using the following formula: percentage inhibition = (1 – sample MFI / MFI negative control) x 100.

**Mucosal and Plasma Cytokine and Chemokine Quantification:** Longitudinal frozen plasma and nasal fluids were analyzed for 41 cytokines/chemokines levels using a human cytokine/chemokine magnetic bead panel HCYTMAG-60K-PX41 Millipore Sigma (Merck Millipore). Nasal fluid was concentrated before the assay and total protein quantified for normalization across biospecimens. Cytokines, chemokines, and growth factors were quantified using on a Luminex 200 Multiplexing Instrument (Luminex, Merck Millipore). The Luminex assay was performed according to the manufacturer’s instructions using duplicate technical replicates. Data obtained were extracted using xPONENT software. The Absolute quantity of cytokines detected was reported as pg/mL of plasma or as pg/mg protein of nasal fluid.

**Statistical Analysis:** The demographic, disease symptoms, comorbidities and treatment characteristics of study participants were described using the number and percent of available data for participants in the study by COVID-19 severity. The age of the cohort described using the median and interquartile range (defined by the 75^th^ percentile). GraphPad Prism software was used for basic statistical analysis: Log-rank Mantel-Cox test for Ab conversion rates, one-way ANOVA with Fisher’s least significant difference (LSD) procedure, followed by a posttest for linear trends for mean differences in Ab magnitude, and two-way ANOVA with Tukey's honestly significant difference (HSD) test for mean differences in cytokine expression among outcome groups over time. XLSTAT package was used for expression analysis, heatmaps, and correlation analysis. Differential expression analysis was performed using XLSTAT analysis set to parametric test type, and Tukey (HSD). Correlations between the different parameters were calculated using the Pearson’s correlation test in XLSTAT and were plotted using OriginPro. Asterisks symbolize p values from these analyses as follows: p < .05 (*), p < .01 (**), p < .001 (***), and p < .0001 (****).

**Supplementary References for Methods**

E1. Chu DKW, Pan Y, Cheng SMS, Hui KPY, Krishnan P, Liu Y, Ng DYM, Wan CKC, yang P, Wang Q, Peiris M, Poon LLM. Molecular Diagnosis of a Novel Coronavirus (2019-nCoV) Causing an Outbreak of Pneumonia. *Clin Chem* 2020; 66: 549-55.

E2. Zhu X, Chang TC, Webby R, Wu G. idCOV: a pipeline for quick clade identification of SARS-CoV-2 isolates. *bioRxiv* 2020.10.08.330456 [preprint]. 2020 Oct 9. Available from https://doi.org/10.1101/2020.10.08.330456.

E3. Whitt MA. Generation of VSV pseudotypes using recombinant ΔG-VSV for studies on virus entry, identification of entry inhibitors, and immune responses to vaccines. *J Virol Methods* 2010; 169: 365-74.

E4. Amanat F, Stadlbauer D, Strohmeier S, Nguyen THO, Chromikova V, McMahon M, Jiang K, Arunkumar GA, Jurczyszak D, Polanco J, Bermudez-Gonzalez M, Kleiner G, Aydillo T, Miorin L, Fierer DS, Lugo LA, Kojic EM, Stoever J, Liu STH, Cunningham-Rundles C, Felgner PL, Moran T, García-Sastre A, Caplivski D, Cheng AC, Kedzierska K, Vapalahti O, Hepojoki JM, Simon V, Krammer F. A serological assay to detect SARS-CoV-2 seroconversion in humans. Nat Med 2020; 26: 1033-6.

E5. Lefrancois L, Lyles DS. The interaction of antibody with the major surface glycoprotein of vesicular stomatitis virus. I. Analysis of neutralizing epitopes with monoclonal antibodies. *Virology* 1982; 121: 157-67.

E6. Matsuyama S, Nao N, Shirato K, Kawase M, Saito S, Takayama I, Nagata N, Sekizuka T, Katoh H, Kato F, Sakata M, Tahara M, Kutsuna S, Ohmagari N, Kuroda M, Suzuki T, Kageyama T, Takeda M . Enhanced isolation of SARS-CoV-2 by TMPRSS2-expressing cells. *Proc Natl Acad Sci U S A* 2020; 117: 7001-3.

E7. Garcia-Beltran WF, Lam EC, Astudillo MG, Yang D, Miller TE, Feldman J, Hauser BM, Caradonna TM, Clayton KL, Nitido AD, Murali MR, Alter G, Charles RC, Dighe A, Branda JA, Lennerz JK, Lingwood D, Schmidt AG, Iafrate AJ, Balazs AB. COVID-19-neutralizing antibodies predict disease severity and survival. *Cell* 2021; 184: 476-88.e11.

**Supplementary Figure Legends**

**Figure S1. SARS-CoV-2 viral and infection stage characterization.** Viral RNA measured in swabs using quantitative real time RT-PCR (qRT-PCR) in terms of Cycle threshold (Ct) value and viral antigen in NRF and plasma by ELISA. Each circle represents a patient colored by severity: mild (
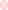
), moderate (●), and severe (●). Positivity thresholds are represented with dotted lines. (A) Viral RNA in nasal swabs over time (top) and between acute (≤ 7 days from diagnosis) and recovery (≥ 8 days from diagnosis; bottom) phase. Viral RNA was isolated from swabs and quantified by qRT-PCR using SARS-CoV-2 specific primers. Ct values were plotted by time from diagnosis. Ct threshold was 40 for PCR positive samples and were plotted by severity groups. Ct values between severity groups mild, moderate, and severe COVID-19 during different time-intervals were compared by one-way ANOVA followed by Fisher’s LSD (**p* ≤ .05; ***p* ≤ .01; ****p* ≤ .001). (B) Viral RNA sequencing data from PCR positive swabs (*n =* 52) plotted by month and percent of SARS-CoV-2 alpha sub-variants each month from July 2020–Jan 2021. The circle size reflects the relative abundance of each sub-lineage per month, and the color scale indicates the number of distinct sub-lineages detected. (C) SARS-CoV-2 specific nucleocapsid and spike proteins in nasal rinses (top and middle) and spike protein in plasma (bottom), respectively, and grouped by disease severity. Severity groups were compared using Kruskal-Wallis Test, a non-parametric alternative to ANOVA, and Dunnet’s test. (D) Severe COVID-19 patients dominated Ag-specific IgM+IgG+ sero-phenotype within a week of SARS-CoV-2 infection. Percent of IgM-IgG-, IgM+IgG- and IgM+IgG+ SARS-CoV-2 specific antibody phenotype in patients were grouped by disease severity and duration (weeks).

**Figure S2. Mucosal and systemic S and N-specific antibody production significantly altered by disease progression and week from diagnosis.** Ag-specific IgA, IgG, and IgM antibodies in nasal rinse fluids and plasma were quantified by ELISA over time following COVID-19 diagnosis. Antibodies were quantitated in the NRF collected longitudinally from participants with mild (*n =* 102 samples), moderate (*n =* 77 samples), and severe (*n =* 23 samples) disease severity and in the plasma samples from participants with mild (*n =* 86 samples), moderate (*n =* 62 samples), and severe (*n =* 23 samples) disease severity. Each circle represents a subject and their classification by COVID-19 severity outcome of mild (●), moderate (●), and severe (●). Study day was converted to time from positive diagnosis (A, B) and grouped by week (C, D). Nasoconversion (A) and seroconversion (B) rates were compared by Log-rank Mantel-Cox test. Mucosal (C) and systemic (D) antibody levels were compared within severity groups overtime. Antibody levels by week from diagnosis were log transformed and analyzed using one-way ANOVA followed by Tukey MCT, asterisks indicate significant difference in the mean. Post test for linear trends was performed and p values for significant trends inset.

**Figure S3. Mucosal and systemic cytokines expression.** Immune factors were quantified on the Luminex® xMAP™ system with a MILLIPLEX MAP human cytokine/chemokine immunoassay. Mucosal and systemic cytokine responses were quantified in 190 NRF samples and 169 plasma samples. (A) Immune factors were grouped by major immune functions: chemoattractants (green), growth factors (red), pro inflammatory (purple), adaptive immunity (blue), and anti-inflammatory (orange).

**Figure S4. Enhanced mucosal with diminished systemic cytokine targeting of dendritic, NK, and T cells is associated with milder COVID-19 progression.** Immune factors were quantified by Luminex during the acute early response phase of COVID-19 (≤7 4 days from diagnosis). The mean pg/mg (mucosa) or pg/ml (plasma) of each immune factor was inputted into the model. The model automatically calculates the magnitude of the flow origin (i.e., the total cytokine input per severity group) and generates node height and flow thickness proportional to picograms of each immune factor. The severity group nodes were colored by type: mild (pink), moderate (salmon), and severe (maroon). The immune factor nodes and chords were shaded by major function: growth factors (red), chemokines (green), anti-inflammatory (orange), adaptive immunity (blue) and pro-inflammatory (purple). Immune factors were linked to their major cellular targets in the context of viral infection. Cell target nodes were colored coded into non-immune cells (yellow), innate immune cells (red), and adaptive immune cells (blue). The flow splits from each immune factor to cell target with node height of each cell type proportional to the total pg of immune factors directed at that cell target. The early response phase (0-4d) mean pg/mg of mucosal immune factors were derived from NRF collected from mild (n = 13), moderate (n = 17) and severe (n = 5) cases (A) and plasma collected from mild (n = 14), moderate (n = 16), and severe (n = 6) cases (B).

**Figure S5. Correlation plots for mucosal and systemic immune factors responses during acute and recovery phase.** The Pearson’s correlation analysis plots for mucosa and plasma immune factors measured in the (A) acute and (B) recovery phases (≤7 days and ≥8 days from diagnosis).

**Figure S6. Significant differences in mucosal and systemic immune factor dynamics are associated with varying degrees of COVID-19 disease severity in recovery.** Quantitative data for NRF and plasma from 41 cytokines, chemokines and growth factors and N and S specific IgA, IgM, and IgG were analyzed. Study day was converted to time from positive diagnosis, >14 to ≤21 days (A) and > 21 days (B). Significant fold changes between patients who developed severe COVID-19 were determined compared to those with mild illness (top panel) and those with moderate COVID-19 (middle panel). Significant fold changes in immune factors between non hospitalized participants with asymptomatic or mild symptoms (mild) were determined compared to those with moderate illness (bottom panel). Differential expression analysis was performed using XLSTAT analysis set to parametric test type, and Tukey (HSD). Volcano plots were graphed using OriginPro. *X-axis* represents log2 the fold change of protein with dashed lines intersecting at the two-fold cutoff point. The *Y-axis* represents -log10 *p* value with dashed lines intersecting at the cut-off for significance (*p* < .05). Proteins that significantly increased ≥ 2-fold are colored red, significantly decreased ≥ 2-fold are colored green, and significantly different proteins with fold changes between -2 and +2 are colored orange. Mucosal (m) and systemic (s) compartments are indicated before protein symbols.

**Figure S7. COVID-19 induces differential expression of immune factors in children and African Americans.** Quantitative data for NRF and plasma from 41 cytokines, chemokines and growth factors and N-Ag and S-Ag specific IgA, IgM, and IgG were analyzed. (A) Outpatient children (*n =* 17 samples) were compared to outpatient adults (*n =* 132 samples), outpatient females (*n =* 81 samples) were compared to outpatient males (*n =* 72 samples) and outpatient African Americans (n = 08 samples) were compared to outpatient Americans of European descent (n = 62 samples). (B) Hospitalized children (n = 24 samples) were compared to hospitalized adults (n = 30 samples), hospitalized females (n = 51 samples) were compared to hospitalized males (n = 18 samples) and hospitalized African Americans (n = 46 samples) were compared to hospitalized Americans of European descent (n = 23 samples). Differential protein expression was determined by Tukey’s HSD test using XLSTAT and plotted using OriginPro software. *X-axis* represents log2 the fold change of protein in the compared groups, and the *Y-axis* represents -log10 *p* value of each Immune factor. The dashed line intersecting the *Y-axis* represent the cut-off for significance (*p* < 0.05). Dashed lines intersecting the *X-axis* represent the two-fold cutoff for increase or decrease protein levels. Proteins that significantly increased ≥ 2-fold are colored red, significantly decreased ≥ 2-fold are colored green, and significantly different proteins with fold changes between -2 to -1.8 and +1.8 to +2 are colored orange.
